# An Understanding of the Global Status of Major Bacterial Pathogens of Milk Concerning Bovine Mastitis: A Systematic Review and Meta-Analysis (Scientometrics)

**DOI:** 10.3390/pathogens10050545

**Published:** 2021-04-30

**Authors:** Paramanandham Krishnamoorthy, Kuralayanapalya P. Suresh, Kavitha S. Jayamma, Bibek R. Shome, Sharanagouda S. Patil, Raghavendra G. Amachawadi

**Affiliations:** 1 Pathoepidemiology Laboratory, ICAR-National Institute of Veterinary Epidemiology and Disease Informatics (NIVEDI), Post Box No. 6450, Ramagondanahalli, Yelahanka, Bengaluru 560064, India; krishvet@gmail.com (P.K.); sureshkp97@rediffmail.com (K.P.S.); kavithaentomology@gmail.com (K.S.J.); brshome@gmail.com (B.R.S.); sharanspin13@gmail.com (S.S.P.); 2 Department of Clinical Sciences, College of Veterinary Medicine, Kansas State University, Manhattan, KS 66506, USA

**Keywords:** *Staphylococcus* species, *Streptococcus* species, *Escherichia coli*, prevalence, world, systematic review, meta-analysis

## Abstract

In this study, the major mastitis pathogen prevalence in the cattle and buffalo of the world was estimated by a meta-analysis. *Staphylococcus* (S) species, *Streptococcus* (St) species, and *Escherichia coli* (Ec) prevalence studies reported during 1979–2019 were collected using online databases, and offline resources. A meta-analysis of these data was done with the meta package in R-Software. The *Staphylococcus aureus* was the major mastitis pathogen, mostly causing subclinical mastitis, Ec causing clinical mastitis and St causing subclinical and clinical mastitis. The pooled prevalence estimates of S, St, and Ec were 28%, 12%, and 11% in the world from 156, 129, and 92 studies, respectively. The S, St, and Ec prevalences were high in Latin America (51%), Oceania (25%), and Oceania (28%), respectively. Higher S, St, and Ec prevalences were observed by molecular methods, signifying high sensitivity and usefulness for future studies. Among bacterial species, *S. aureus* (25%) followed by coagulase-negative *Staphylococcus* species (20%), *Escherichia coli* (11%), *St. agalactiae* (9%), *St.* *uberis* (9%) were the important pathogens present in the milk of the world. We hypothesize that there is a urgent need to reduce mastitis pathogen prevalence by ensuring scientific farm management practices, proper feeding, therapeutic interventions to augment profits in dairying, and improving animal and human health.

## 1. Introduction

Milk is considered to be a staple food for human beings throughout the world, and it also poses a public health risk only when consumed unpasteurized because it contains a high bacterial count and is a good medium for bacterial growth. Bovine mastitis, a century-old production disease caused by various bacteriological agents, has been intensively monitored since 1917 [1]. Mastitis is a disease of dairy cows worldwide [2] and comprises different types, namely subclinical and clinical mastitis. It is caused by multi-etiological pathogens in that bacterial species are considered to be the most important causative agent that leads to milk production loss. Costello [3] found that the annual economic loss due to mastitis was estimated to be US$200 per cow per year. The economic losses due to both subclinical and clinical mastitis are US$98,228 million or 7165.51 crore Indian rupees annually in India, as reported in the literature [4]. A meta-analysis is an innovative tool for estimating the prevalence of various livestock diseases [5]. Lately, livestock disease prevalence estimates have been reported for subclinical and clinical mastitis, major mastitis pathogens in India [6], anaplasmosis in the world [7], and *Staphylococcus aureus* with methicillin-resistance in livestock in India [8]. The foremost reasons doing a meta-analysis is to sum up and amalgamate the results from a number of previous studies. It also aids in investigating the requirement of larger sample sizes, snowballing the accuracy in prevalence estimates, deciding whether new studies are required, and creating new hypotheses for future studies [7,8]. The important concepts to consider while doing a meta-analysis are the selection of studies, heterogeneity in reported prevalence values, and used data analysis methods [9]. 

The cattle and buffalo population in the world were 1489 and 206 million, respectively, in 2018, as reported by the Food and Agricultural Organization (FAO), Rome. The United States of America (USA) produced 82 million tons of cattle milk in 2018 and ranked first in the world, followed by India with 47 million tons; India ranked first in the total (cattle and buffalo) milk production in the world [10]. The per capita consumption of milk was highest in Belarus (111.09 kg) in 2018 [11]. In the world, India ranks first for both buffalo milk production (55 million tons) and buffalo population. In 2018, Brazil had 193.5 million cattle, making it first ranked in the world, while India ranked second [10]. In India, the cattle and buffalo populations were 192.5 and 109.8 million, respectively, out of a total livestock population of 535.8 million in 2019, as reported in the 20th Livestock Census report. In 2019, the per capita availability of milk was 394 grams per day, and 187.75 million tons comprised the total milk production, as reported by the Ministry of Fisheries, Animal Husbandry, and Dairying, Government of India, New Delhi [12]. The major pathogens causing mastitis could be attributed to *Staphylococcus aureus, Streptococcus agalactiae, Corynebacterium bovis, Mycoplasma* species, *Streptococcus uberis* [13], coliforms (*Escherichia coli, Klebsiella* species, and *Enterobacter aerogenes*), *Serratia, Pseudomonas, Proteus* species, environmental *Streptococci*, and *Enterobacter* species (as described earlier) [14]. Amongst the bacterial species, *Staphylococcus* (S), *Streptococcus* (St), and *Escherichia coli* (Ec) have been deemed as the major mastitis pathogens in dairy cattle and buffalo. Numerous studies are available on S, St, and Ec prevalence in the milk of cattle and buffalo reported from various geographical locations in several countries of the world, which have indicated highly conflicting results regarding the major mastitis pathogen prevalences. Our previous study analyzed and reported the major mastitis pathogen prevalences in India from 1995 to 2016 by using a meta-analysis [6]. However, there have been no studies on the prevalence status of three major mastitis pathogens in the world. Keeping that in mind, the present study was undertaken to identify the highly important mastitis pathogens and their prevalence status among the three major mastitis pathogen groups in the milk of cattle and buffalo in the world by using systematic review and a meta-analysis.

## 2. Results

### 2.1. Staphylococcus (S) Species, Streptococcus (St) Species, and Escherichia coli (Ec) Prevalence Studies

**Figure 1:**
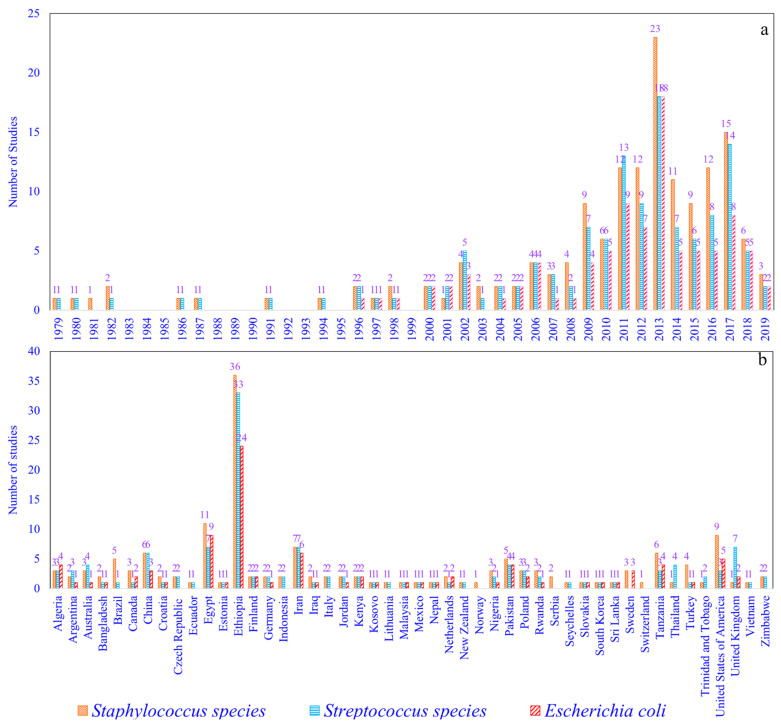
Year-wise (**a**) and country-wise (**b**) number of prevalence studies from the world included for meta-analysis.

The systematic review and meta-analysis of S, St, and Ec prevalence studies in cattle and buffalo from the world were undertaken in the present study. The filled-in PRISMA checklist for systematic review and meta-analysis is given in Supplementary File S1. The number of studies on S, St, and Ec prevalence in milk for the world included for meta-analysis were 156, 129, and 92, respectively, and they included studies from all continents except for Antarctica. The details of the studies regarding the continents of the world; countries; author name; year; type of mastitis; reported prevalence of S, St, or Ec; and quality assessment scores are given in Table 1. The number of countries included for determining S, St, and Ec prevalence estimates were 49, 45, and 34, respectively. There were more prevalence studies from Europe (16) than from Oceania (2). There were more prevalence studies for *S. aureus* (137) and *St. agalactiae* (83) than the other bacterial species. The number of studies reported based on year and country for the world is given in Figure 1. The S, St, and Ec prevalence studies reported during the periods of 1979–2019, 1979–2019, and 1996–2019, respectively, from the world were included for meta-analysis. The increased number of studies reported were 23 (S), 18 (St), and 18 (Ec) during the year 2013. Ethiopia reported the highest number of studies on S, St, and Ec prevalence, with 36, 33, and 24 studies, respectively. The S, St, and Ec prevalence studies from India were analyzed separately, as mentioned earlier, and an attempt was made as an example for better understanding. 

The details of the meta-regression analysis of the different characteristics of S, St, and Ec prevalence studies are given in Table 2. The S prevalence studies from the world revealed significant (*p* < 0.01) predictors as continents, methods used for identification, different bacterial species, countries, and St prevalence the year; methods and countries were highly (*p* < 0.01) significant. The methods used, host, and countries were revealed to be significant predictors for Ec prevalence estimates. 

### 2.2. Prevalence of S, St, and Ec in the World

**Figure 2:**
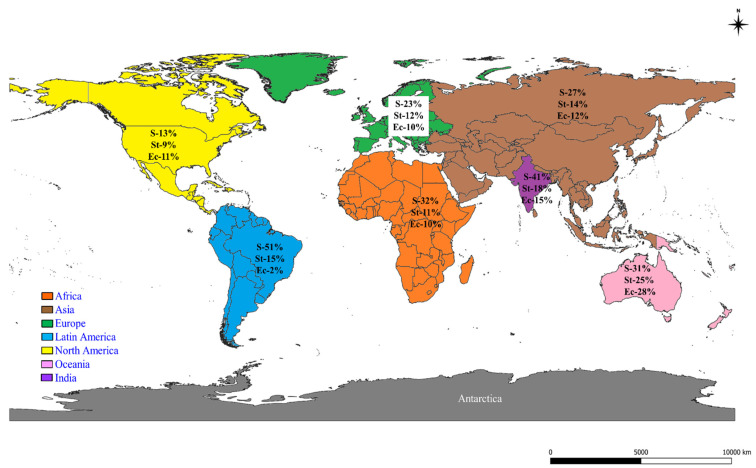
World map showing the continent-wise prevalence of *Staphylococcus* species (S), *Streptococcus* species (St), and *Escherichia coli* (Ec).

The particulars of S, St, and Ec prevalence in the world acquired by meta-analysis are shown in Table 3, Table 4 and Table 5, respectively. The pooled prevalence estimates for S, St, and Ec were 28%, 12%, and 11%, respectively, obtained from 283,685, 272,539, and 257,473 samples, respectively, from six continents of the world. The major mastitis pathogen prevalence appeared to be increasing in recent years, i.e., 2011–2019, in contrast to the past periods. Continent-wise S, St, and Ec prevalence estimates are depicted as a map in Figure 2. High S, St, and Ec prevalences were observed in Latin America (51%), Oceania (28%), and Oceania (25%), respectively. The period-wise analysis revealed an increasing prevalence of S in Africa, North America, Oceania, as well as a decreasing trend in Asia, Europe, and Latin America during the 2011–2019 period. St showed an increasing prevalence in recent times, i.e., 2011–2019. A higher Ec prevalence during 2011–2019 was observed in Africa and Asia, and a decreasing prevalence was found in Europe and North America. The S, St, and Ec prevalences found by molecular methods were 35%, 17%, and 23%, respectively. The bacterial species-wise analysis revealed a higher prevalence of *S. aureus* (25%), followed by *E. coli* (11%), *S. epidermidis* (9%), *St. agalactiae* (9%), and *St. uberis* (9%). A higher prevalence of *Staphylococcus* species in subclinical mastitis, *Streptococcus* species in both forms of mastitis, and *Escherichia coli* in clinical mastitis were noticed. Regarding the test for publication bias, the *p*-value obtained for overall prevalence estimates for S, St, and Ec were 0.410, 0.586, and 0.219, respectively, specifying that there was no bias among the prevalence studies. The Cochran Q statistics showed highly significant (*p* < 0.01) differences between the studies included for S, St, and Ec prevalence in the world sub-grouped based on year, continent, period, species, method, bacterial species, and type of mastitis, showing the heterogeneity between the studies selected for meta-analysis. There were few exceptions noticed, such as a significant (*p* < 0.05) difference for *St. acidominimus* and no significant difference for *S. captis, S. cohni, S. kloosi, S. lentus, St. anginosus, St. bovis, St. intermedius*, and *St. mitis*, as well as the North America period II for both in St and Ec that denoted no heterogeneity among the considered studies.

### 2.3. The S, St, and Ec Prevalence in Various Countries

**Figure 3:**
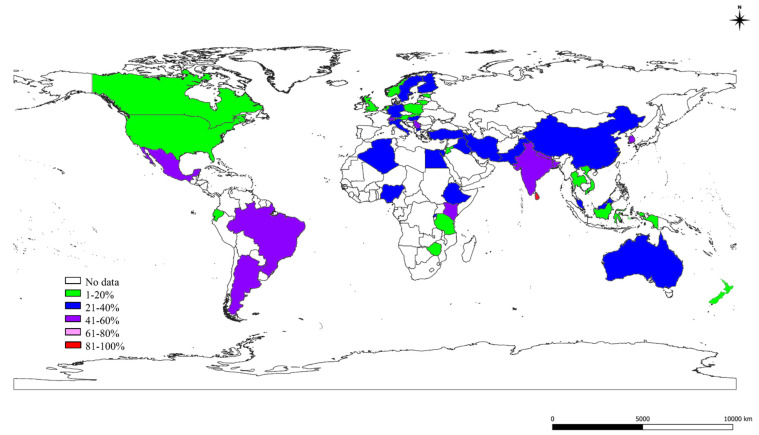
World map showing the prevalence estimates of *Staphylococcus* species in different countries.

**Figure 4:**
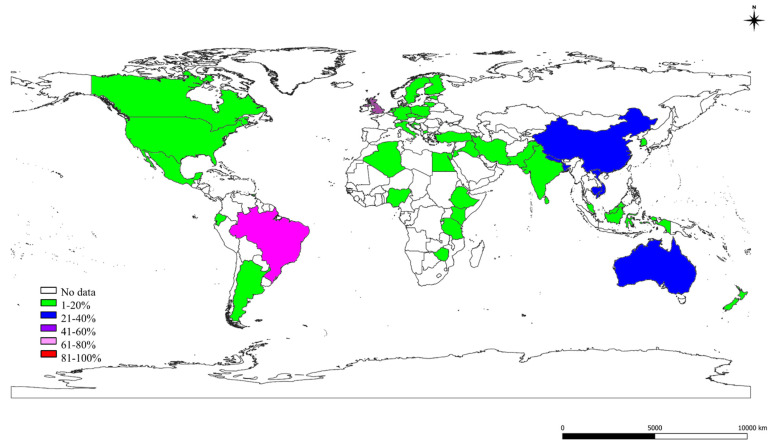
World map showing the prevalence estimates of *Streptococcus* species in different countries.

**Figure 5:**
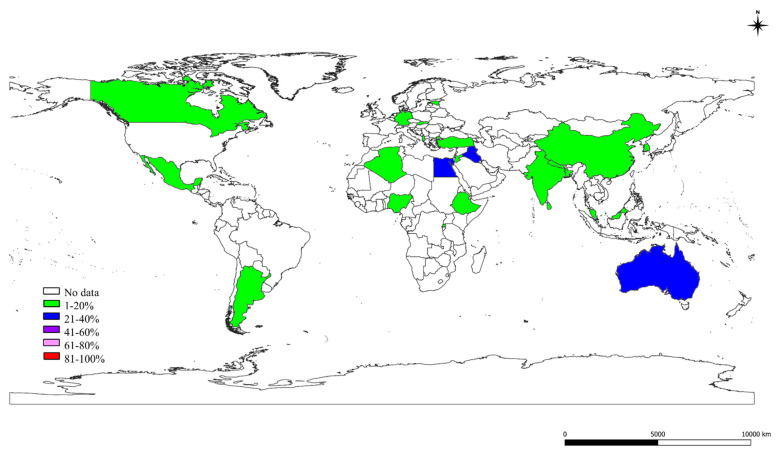
World map showing the prevalence estimates of *Escherichia coli* in different countries.

The details of S, St, and Ec prevalence estimates obtained for various countries of the world analyzed by meta-analysis are presented in Figure 3, Figure 4 and Figure 5, respectively. The S prevalence was highest in Trinidad and Tobago (98%), followed by Sri Lanka (90%); in other countries, a low prevalence was observed in Vietnam (5%). A higher St prevalence was observed in Brazil (67%), followed by the United Kingdom (49%); there was a low prevalence in other countries like Jordan (1%), Tanzania (2%), Zimbabwe (3%). The Ec prevalence was high in Australia (28%) and Iraq (24%), and it was low in Croatia and Rwanda (1%), as shown in Figure 5. The countrywide S prevalence studies showed a highly significant (*p* < 0.01) difference based on Cochran Q statistics for the various countries except for Canada, Iraq, Kenya, and Netherlands with a significant (*p* < 0.05) difference, and there was no significant difference for Algeria, Indonesia, and Italy—specifying no heterogeneity in studies. A highly significant (*p* < 0.01) difference for Cochran Q values for St prevalence studies was observed except for Germany; Nigeria revealed a significant (*p* < 0.05) difference, and Algeria, Czech Republic, Italy, Jordan, and Kenya showed no significant differences. 

**Figure 6:**
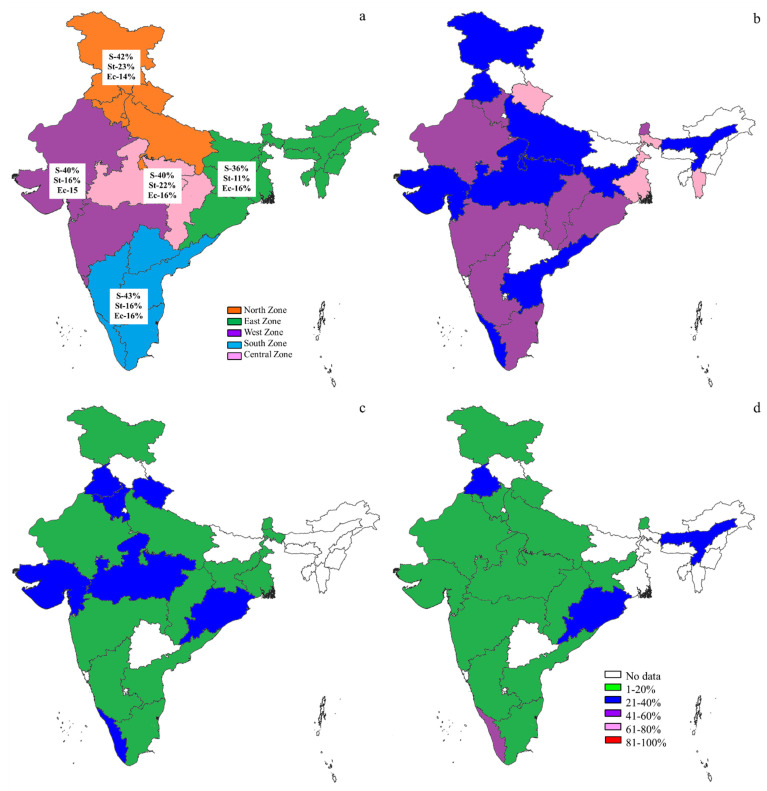
Zone-wise (**a**) and state-wise prevalence of *Staphylococcus* species (**b**), *Streptococcus* species (**c**), and *Escherichia coli* (**d**).

The particulars of S, St, and Ec prevalence estimates obtained for India based on period, zone, state, species, method, bacterial species, and type of mastitis are shown in Supplementary File S2. The pooled prevalence estimates of S, St, and Ec in India were 41%, 18%, and 15%, respectively, obtained from 14,011, 12,314, and 12,288 milk samples. The details of zone-wise and state-wise prevalence estimates of S, St, and Ec in India are shown in Figure 6. Higher S, St, and Ec prevalences in the south zone (43%), north zone (23%), and east and south zones (16%), respectively, were observed. The state-wise breakdown revealed the highest S, St, and Ec prevalences in Mizoram and Uttarakhand (67%), Odisha (32%), and Kerala (42%), respectively. Low prevalences of S, St, and Ec were observed in Jharkhand (21%), Sikkim (3%), and Uttarakhand (3%), respectively. The publication bias *p*-values estimated by Begg’s test were 0.609, 0.923, and 0.869 for S, St, and Ec, respectively, which implied no publication bias among the studies from India included for obtaining pooled prevalence estimates.

## 3. Discussion

Bovine mastitis is considered to be a hundred-year-old production disease [1] and still challenging to prevent, even with advances in technological interventions for diagnosis and treatment, mainly due to the multi-etiological nature of the disease. The presence of bacterial species in raw milk affects not only the udder health of dairy cattle and buffalo but also human health by the possible transmission of antimicrobial resistance. The meta-analysis has been gaining importance in recent years in determining livestock disease prevalence estimates, as described earlier [5]. The S species, St species, and Ec prevalences were found to be 28%, 12%, and 11%, respectively, in the world based on the current meta-analysis. However, a study from Iran reported a higher prevalence of S species in mastitis—71.5% in milk [15]—in comparison to the present study. The *Staphylococcus aureus* is considered to be the main mastitis pathogen among the three pathogens in the world based on the present study, which was in agreement with previous reports from Norway, Australia, the United States of America, Italy, Tanzania, and Finland, as well as a 100-year review on bovine mastitis [1,16,17,18,19,20,21]. The prevalence of coagulase-negative *Staphylococcus* (CNS) species obtained in the present study agreed with a previous study that indicated the prevalences of subclinical and clinical mastitis at 6–72% and 6–30%, respectively [22].

The importance of CNS species in mastitis cases in Iran was previously described [15]. The pathogenic microorganisms enter through the teat canal and result in physical, chemical, and pathological changes in the udder and milk [13,14]. The foremost factors responsible for the occurrence of mastitis may be determined by the exposure to microorganisms, the immune mechanism of dairy cows, managemental factors, and environmental factors, as described in [14]. The environment plays an important role in the spread of mastitis pathogens in dairy cattle, e.g., the horn flies reported in [18]. In a previous study, *S. aureus* was isolated from heifer body sites, environments, human beings, and horn flies in a farm, and similar or identical genotypes were isolated from the cow milk samples [18], suggesting that S species infection predominantly comes from the environment (except for *S. aureus*) and mainly causes subclinical mastitis. Furthermore, there were more studies in more countries on S prevalence than St and Ec, which confirms the significance of S species in various countries of the world. 

The number of countries showing a higher S prevalence estimate was 41 whereas, the higher prevalence of St in 6 and Ec in 2 countries were observed. This finding was crucial for showing S species as important mastitis pathogen sources in many countries of the world. A higher St prevalence was noticed in Brazil, China, New Zealand, Thailand, the United Kingdom, and Vietnam, and a higher Ec prevalence was noticed in Canada and Sri Lanka—suggesting the significance of St and Ec as the principal mastitis pathogens in these countries. A year-wise evaluation revealed that the S and St species increased during 2011–2019 in comparison to past years, which may have been due to advances in the diagnosis of intramammary infections in bovine populations [21,23] and the use of molecular methods for bacterial identification. A continent-wise analysis showed a higher prevalence of S in Latin America and higher prevalences of St and Ec in Oceania, which was corroborated with the earlier studies [24,25]. However, more studies were reported from Africa, signifying the importance of the mastitis problem in the countries of such. This might because of the low economic status, poor husbandry practices, and dairy management practices of African countries. The countries in Latin America predominately have grass-dependent production systems, and the risk of exposure to environmental pathogens is greater than in other countries where cows are housed in sheds, as described in [24]. The worldwide St species prevalence was found to be 12%, and the most common bacterial species among them were *St. agalactiae* and *St. uberis* (9% each), which agreed with a previous study that indicated *St. uberis* as a common environmental bacteria causing clinical mastitis and infecting dairy heifers from a pasture grazing rearing system in New Zealand [25]. Among cattle and buffalo, higher S and Ec prevalences were observed in buffalo, and a higher St prevalence was observed in cattle. The higher prevalence of S species in buffalo was reported earlier in a study from Pakistan [26], which agreed with the present study. Furthermore, regarding the diagnostic methods employed for the identification of mastitis pathogens, the molecular methods revealed higher prevalence estimates for all three pathogens. This signified the higher sensitivity of molecular methods in the identification of bacterial pathogens isolated from milk in dairy cattle and buffalo, and these methods may be used in future studies. However, the majority of the studies employed cultural examination and biochemical tests for the confirmation of bacterial species isolated from milk samples in various countries. This might have been because isolation and identification via cultural examination and biochemical tests are easier and more cost-effective for bacterial species identification than molecular methods, which are usually costly. Among the S and St species, *S. aureus, St. agalactiae*, and *St. uberis* revealed higher prevalences, suggesting the importance of these species in mastitis, which is in agreement with the previous studies [18,24,25]. In a study conducted in Nigeria with mastitis cows, the *Streptococci* species was considered to be an environmental pathogen accountable for a high proportion of mastitis cases, mainly with *St. uberis* [27]. Furthermore, there was a considerable prevalence of *St. dysgalactiae* in mastitis cases in the dairy herds of China [28], as corroborated by the present study. Higher S, St, and Ec prevalences were observed in subclinical mastitis and clinical mastitis. The S species are more crucial in subclinical mastitis than in clinical mastitis, mostly contributing as contagious (especially *S. aureus* and environmental pathogens) and leading to production loss without any clinical signs and was in agreement with previous reports [19,22]. However, Ec has a greater chance of causing severe, acute mastitis before forming clinical mastitis cases compared to other bacterial pathogens. Coliform organisms were reported to cause severe clinical mastitis in cattle and buffalo [13], in agreement with the present study. Among the three major pathogens, the St species was present in both the forms of mastitis, i.e., subclinical and clinical mastitis, in dairy cattle and buffalo of the world. The mastitis caused by these microorganisms can be prevented by following proper husbandry practices such as using proper milking systems, engaging in hand disinfection before milking, milking affected cows at the end, conducting frequent testing for mastitis, appropriately using dry cow therapy, and removing chronically infected dairy cows from the farm as necessary [29]. The geographical location of the farm, used bedding materials, and season may be considered while planning mastitis control and prevention strategies in dairy farms, as mentioned earlier [30]. Furthermore, clean and safe milk production is important for the health of humans as a whole because milk forms an essential part of food in almost all the countries of the world.

In India, the prevalences of S, St, and Ec were reported to be 41%, 18%, and 15%, respectively, and the major mastitis pathogen was the S species (similar to the rest of the world). A previous study on major mastitis pathogen prevalences in dairy cattle in India reported 45%, 13%, and 14% for S, St, and Ec, respectively, based on a meta-analysis of studies from the limited period of 2005–2016 [6]; this was corroborated by the present study, which included studies reported during 1995–2019. The findings from the present study were in agreement with the previous reports that indicated the S species as a major mastitis pathogen of subclinical mastitis in cows [31,32,33]. Based on a zone-wise analysis, higher S, St, and Ec prevalences were observed in the south, north, and east and south zones, respectively. As reported here, the highest prevalence of S was in Uttarakhand and Mizoram (67%), highest prevalence of St was in Odisha (32%), and Ec prevalence was highest in Kerala (42%) based on state-wise analysis. The S, St, and Ec prevalences were lowest in Jharkhand, Sikkim, and Uttarakhand, respectively. It was shown that there were variations in the occurrence of mastitis pathogens in different states, and it is necessary to take up preventive and control measures accordingly. The variations in the mastitis pathogen prevalence might be due to differences in the management, feeding, and husbandry practices among the states in India. S and Ec occur due to unclean milking practices, contaminated environments, dairy farmworkers and the pathogens enter through the teat canal, causing infection in the udder as reported earlier [34]). Cattle showed a higher prevalence of St and Ec than buffalo, but the S prevalence was higher in buffalo. Furthermore, a comprehensive examination of milk could offer important evidence concerning the nature of mastitis in dairy cattle and buffalo [35]. Bacterial species-wise prevalence estimates in this study were higher for *S. aureus* (38%) and *St. dysgalactiae* (15%), which were in agreement with a previous report [36]. Moreover, *S. aureus* in milk leads to food poisoning in human beings and is considered a public health concern. Further, an *S. aureus* presence in milk and milk products indicates poor quality [36]. *St. dysgalactiae* was considered a contagious mastitis pathogen in developed countries, namely the USA and UK, as described earlier [37,38]. In India, Ec was observed to be second in prevalence following S species, which is in contrast to the many reports [39,40,41]. According to the type of mastitis, S was prevalent in subclinical mastitis and St and Ec were present in both subclinical and clinical mastitis; similar observations were previously reported [33,38]. This also confirms that S species cause a subclinical and chronic form of mastitis and that Ec leads to a clinical and acute form of mastitis in dairy cattle and buffalo.

As this report attempted to estimate the worldwide prevalence of mastitis pathogens for the first time and to find the major worldwide mastitis pathogens, a few limitations must be disclosed. The prevalence estimates did not include the association of mastitis occurrence with various risk factors. The risk factors included the cattle and buffalo breeds, genetic character of the breed, lactation stage, number of lactations, milk yield or production, followed farm management practices, climatic factors, and the geographical location of the reported studies, all of which may have resulted in variation among the mastitis pathogen prevalence [6,42]. The studies included for meta-analysis were from 49 countries that have reported major mastitis pathogen prevalences and are available in online databases and offline literature, and some countries may have had small numbers of studies but nonetheless gave estimates for the various mastitis pathogens in the world. Further studies on mastitis pathogens are required from other countries to obtain more accurate prevalence estimates. Some of the countries only had a few studies included for meta-analysis and thus may not have given a true picture of the country. The studies were comprehensively collected from different online databases and offline resources in the present study. However, the comprehensiveness of all the studies reported should be investigated in a future meta-analysis because is very important to more precisely determine the prevalence estimates. Many other bacterial species might cause subclinical and clinical mastitis, but in the present study, only three important bacterial pathogens were considered for the world. The studies collated in this report will form a resource on major mastitis pathogen prevalence studies, enabling simple access for the researchers on mastitis in upcoming years.

## 4. Materials and Methods

### 4.1. Literature Search

**Figure 7:**
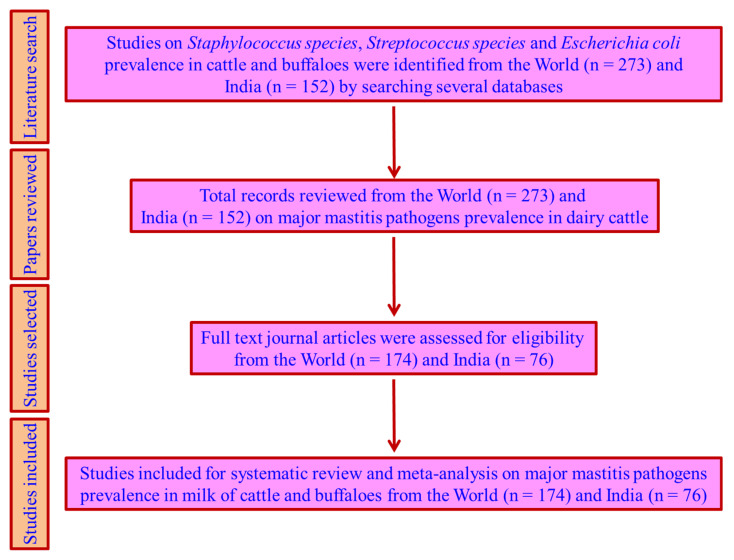
Flow chart showing review and selection of studies for meta-analysis.

The literature search was systematically carried out by using keywords for the identification of prevalence studies on *Staphylococcus* (S) species, *Streptococcus* (St) species, and *Escherichia coli* (Ec) in milk. The preferred reporting items for systematic reviews and meta-analyses (PRISMA) guidelines were followed in the selection of major mastitis pathogen prevalence studies from the world [43]. A flow chart detailing the number of S, St, and Ec prevalence studies retrieved, reviewed in full, and collated for the meta-analysis for the world and India is given in Figure 7. The search terms used were “prevalence of mastitis pathogens, prevalence of *Staphylococcus* species, prevalence of *Streptococcus* species, and prevalence of *Escherichia coli*,” not including the Boolean operators. The online databases searched were Consortium of e-Resources in Agriculture (CeRA) under the Indian Council of Agricultural Research, Elsevier, Google Scholar, Indian journals.com, PubMed, Springer, and Web of science. Around 273 and 152 articles from the world and India, respectively, were identified based on various database searches. The selection of the studies was based on the characteristics of journal articles, including author names, publication year, country, state, number of positive samples, number of samples tested, engaged diagnostic methods, host species (either cattle or buffalo), identified bacterial species, and milk samples from subclinical or clinical mastitis or mastitis in general. Finally, the selected journal articles were reviewed in detail, and the cited references were used for back-searching for pertinent studies. The reported studies included for data extraction were limited to the period from January 1979 to December 2019, and the studies were only written in the English language.

### 4.2. Extraction of Data

The prevalence reports on major mastitis pathogens were thoroughly reviewed and based on fixed inclusion and exclusion criteria encompassed for a meta-analysis. The inclusion criteria comprised both longitudinal and cross-sectional studies reported in the journal articles, mainly with details mentioned below in the characteristics of the studies from the countries of the world, and exclusion criteria were case reports and review articles. The study details were extracted from the characteristics reported in each study into a predesigned format in Microsoft Excel sheets. These characteristics were author names; year of publication; species of animals tested (cattle, buffalo, or both); number of samples positive for S, St, and Ec; the total number of samples tested; prevalences of S, St, and Ec; the applied diagnostic methodologies; and the types of mastitis cases. The particulars of employed diagnostic methods were bacterial cultural examination (e.g., isolation, cultural characters, and biochemical and phenotypic tests), molecular methods (e.g., established on nucleic acid methods), and other methods. Among the employed diagnostic methods and identified bacterial species, the highest prevalence values obtained by a method or a bacterial species in the S and St groups were included for meta-analysis wherever applicable. The data sorting was done based on a three-step methodology, as stated earlier [7].

### 4.3. Quality Assessment of Studies

The quality assessment of the prevalence studies selected for a meta-analysis was done by a fixed rating scale devised in a previous study [44] with some modifications as mentioned below. The rating scale involved the following parameters: sample representation, size of the sample, methods employed for pathogen identification, prevalence values, and assessment of the outcome, with each having a maximum score of 2, 2, 2, 2, and 2, respectively. The maximum score obtained for the quality assessment of the study was 10, and the minimum required score was 5 for the inclusion of the studies in the meta-analysis.

### 4.4. Statistical Analysis

The meta-analysis was performed in R Open source scripting software version 3.2.5 (Comprehensive R Archive Network, Vienna, Austria) by using R package "meta," as reported in [45]. The obtained meta-analysis results were depicted in the form of forest plots, also called confidence interval (CI) plots, which present the estimates for the prevalence with confidence intervals for each study. The maximum likelihood estimation (method.tau = ML) method and logit transformation (sm = PLOGIT) were used for the meta-analysis in this study. The mastitis pathogen prevalence is shown as a square, and the horizontal line extending from either side represents the CI at a 95% level for each study considered. The shaded thick red line given below the forest plot is the prediction interval (PI) at the 95% level. The heterogeneity among the reported studies encompassed in the meta-analysis was determined by Cochran Q test, and the I-Square, Tau square, H, and *p* values were obtained. If the studies indicated heterogeneity based on the Cochran Q (*p* < 0.05) and I-square (>50%) values, the random effects model (Der Simonion and Laird Method) was used to determine the prevalence estimates. The Cochran Q statistics were determined as per the method reported earlier [7,8] and specify the significance level. The publication bias was assessed by using the Begg and Mazumdar adjusted rank correlation test for the overall prevalence estimates, and it is expressed as a *p*-value. A meta-regression analysis was done to determine the characteristics of the large number of studies that influenced the prevalence estimates. The regression analysis included the model results, the test of moderators, and the tau square, I^2^, H^2^, and R^2^ values. Based on the *p*-values obtained in the analysis, the potential moderator or modifier of prevalence estimates was determined. The subgroup analysis was done based on various parameters reported in an earlier study [7], with some modifications given below, to understand the heterogeneity among the studies on S, St, and Ec prevalence in the world. A funnel plot, which was used to determine the outliers and to understand the nature of the selected studies, was also used. The pre-specified subgroups analyses were (i) overall S, St, and Ec prevalence; (ii) year (1970–2000, 2001–2010, and 2011–2019); (iii) continent (Africa, Asia, Europe, Latin America, North America, and Oceania); (iv) period for continents (before 2011 and after 2011); (v) host species (cattle and buffalo); (vii) method (cultural examination, molecular methods, and other methods); (vii) bacterial species; (viii) type of mastitis (subclinical mastitis and clinical mastitis, mastitis); and ix) country for the world. The overall prevalence estimates for S, St, and Ec based on subgroup analyses were expressed as a percentage along with CI and PI at the 95% level.

## 5. Conclusions

In the present study, it was found that the *Staphylococcus* species is the major mastitis pathogen present in the milk of dairy cattle and buffalo in the world, followed by *Streptococcus* species and *Escherichia coli.* Among the bacterial species, *S. aureus* (25%), coagulase-negative *Staphylococcus* species (20%), *Escherichia coli* (11%), *St. agalactiae* (9%), and *St. uberis* (9%) are the most important mastitis pathogens present in the world. Based on the methods employed for the identification of bacterial species, the molecular methods were found to be more sensitive than culture methods and may be considered in future studies. Further, the S species occurs mostly in subclinical mastitis cases, St occurs in both forms of mastitis, and Ec occurs in clinical mastitis cases. The high-risk countries in the world, global zones, and states in India for mastitis pathogens that were recognized in this study will help policymakers and stakeholders to devise the appropriate preventive measures against these mastitis pathogens. There is an urgent need to improve quality and to avoid bacterial infection in milk by following scientific dairy management practices, clean milk production methods, the regular screening of dairy cattle and buffalo for subclinical mastitis, and proper therapeutic interventions based on antibiotic susceptibility testing. This will help to maintain milk quality, thus preventing public health risks and antimicrobial resistance in human beings. Furthermore, prevalence studies of mastitis pathogens are required from the majority of the countries of the world in the future to get more precise estimates.

## Figures and Tables

**Table 1:** Particulars of major mastitis pathogen prevalence studies from the world, with their quality assessment scores, included for a meta-analysis.

No.	Continents	Countries	Studies (Author and Year)	SCM-1, CM-2, M-3	*S.*-1, *St.*-2, *Ec*-3	Quality Assessment of the Studies #					
						Sample Representation (Maximum Score = 2)	Size of Sample (Maximum Score = 2)	Methods Employed (Maximum Score = 2)	Prevalence Values (Maximum Score = 2)	Assessment of Outcome (Maximum Score = 2)	Total Score (Maximum Score = 10)
1.	Africa	Algeria	Akkou et al., 2018	3	1	**	**	*	**	*	8
2.			Bakir et al., 2011	1	1, 2, 3	*	*	*	*	*	5
3.			Benhamed et al., 2011	3	1, 2, 3	**	*	*	**	*	7
4.			Saidi et al., 2013	3	1, 2, 3	**	**	*	**	*	8
5.		Egypt	Abdel-Rady and Sayed, 2009	1	1, 2, 3	**	**	*	**	*	8
6.			Abd-Elrahman, 2013	3	1, 2, 3	*	*	*	**	*	6
7.			Abo-Shama, 2014	3	1	**	**	**	**	*	9
8.			Ahmed et al., 2018	1	3	**	**	**	**	*	9
9.			Amin et al., 2011	3	1, 2, 3	**	**	**	**	*	9
10.			El-Jakee et al., 2013	3	1	**	**	**	**	*	9
11.			Elbably et al., 2013	3	1, 3	**	**	*	**	*	8
12.			Elhaig and Selim, 2014	1	1, 2	**	**	**	**	*	9
13.			Elsayed et al., 2015	3	1	**	**	**	**	*	9
14.			Hamed and Zaitoun,2014	1	1, 2, 3	**	**	**	**	*	9
15.			Lamey et al., 2013	3	3	*	*	*	**	*	6
16.			Sayed et al., 2014	3	1, 2, 3	*	*	*	**	*	6
17.			Zaki et al., 2010	1	1, 2, 3	**	**	*	**	*	8
18.		Ethiopia	Abebe et al., 2012	3	1, 2, 3	**	**	*	**	*	8
19.			Abebe et al., 2016	3	1	**	**	*	**	*	8
20.			Abera et al., 2013	3	1, 2, 3	**	**	*	**	*	8
21.			Abunna et al., 2013	3	1, 2, 3	**	**	*	**	*	8
22.			Adane et al., 2012	3	1, 2, 3	**	**	*	**	*	8
23.			Amin et al., 2017	3	2	**	**	*	**	*	8
24.			Ayano et al., 2013	1	1, 2, 3	**	**	*	**	*	8
25.			Belayneh et al., 2013	3	1, 2, 3	**	**	*	**	*	8
26.			Birhanu et al., 2017	1	1, 2, 3	**	**	*	**	*	8
27.			Dego and Tareke, 2003	3	1, 2	**	*	*	**	*	7
28.			Demme and Abegaz, 2015	2	1, 2, 3	*	*	*	**	*	6
29.			Duguma et al., 2014	3	1, 2	**	*	*	**	*	7
30.			Elemo et al., 2017	3	1	**	**	*	**	*	8
31.			Getahun et al., 2008	3	1, 2	**	**	*	**	*	8
32.			Haftu et al., 2012	3	1, 2, 3	**	**	*	**	*	8
33.			Hailemeskel et al., 2014	3	1, 2, 3	**	**	*	**	*	8
34.			Kedir et al., 2016	3	1	**	**	*	**	*	8
35.			Lakew et al., 2009	3	1, 2	**	**	*	**	*	8
36.			Megersa et al., 2012	3	1, 2, 3	**	**	*	**	*	8
37.			Mekibib et al., 2010	3	1, 2, 3	**	**	*	**	*	8
38.			Mekonnen and Tesfaye, 2010	3	1, 2	**	**	*	**	*	8
39.			Michael et al., 2013	3	1, 2	**	**	*	**	*	8
40.			Mulate et al., 2017	1	1, 2, 3	**	**	*	**	*	8
41.			Pal et al., 2017	1	1, 2, 3	**	*	*	**	*	7
42.			Seid et al., 2015	3	1, 2, 3	**	**	*	**	*	8
43.			Shiferaw and Telila, 2016	3	1, 2	**	**	*	**	*	8
44.			Sori et al., 2005	3	1, 2, 3	*	*	*	**	*	6
45.			Tadesse and Chanie, 2012	3	1, 2, 3	**	**	*	**	*	8
46.			Tekle and Berihe, 2015	3	1, 3	**	**	*	**	*	8
47.			Tesfaye and Albera, 2018	3	1, 2, 3	**	**	*	**	*	8
48.			Tesfaye, 2017	3	1, 2, 3	**	**	*	**	*	8
49.			Wubishet et al., 2013	3	1, 2	**	*	*	**	*	7
50.			Yohannes and Alemu, 2018	3	1, 2, 3	**	**	*	**	*	8
51.			Yohannis and Molla, 2013	3	1, 2, 3	**	**	*	**	*	8
52.			Zenebe et al., 2014	3	1, 2, 3	**	**	*	**	*	8
53.			Zeryehun and Abera, 2017	3	1, 2	**	**	*	**	*	8
54.			Zeryehun et al., 2013	3	1, 2, 3	**	**	*	**	*	8
55.		Kenya	Mureithi et al., 2017	1	1, 2, 3	**	*	*	**	*	7
56.			Ondiek et al., 2013	1	1, 2, 3	**	*	*	**	*	7
57.		Nigeria	Amosun et al., 2010	2	2	*	*	*	**	*	6
58.			Junaidu et al., 2011	3	1, 2, 3	**	*	*	**	*	7
59.			Marimuthu et al., 2014	2	1	**	**	*	**	*	8
60.			Umaru et al., 2017	3	1	**	**	*	**	*	8
61.		Rwanda	Iraguha et al., 2015	1	1	**	**	*	**	*	8
62.			Mpatswenumugabo et al., 2017	1	1, 2, 3	**	**	*	**	*	8
63.			Ndahetuye et al., 2019	1	1, 2	**	**	*	**	*	8
64.		Seychelles	Watson et al., 1996	3	1, 2	**	*	*	**	*	7
65.		Tanzania	Kivaria et al., 2006	3	1, 2, 3	**	**	*	**	*	8
66.			Mdegela et al., 2004	3	1, 2	**	**	*	**	*	8
67.			Mdegela et al., 2009	3	1, 2, 3	**	*	*	**	*	7
68.			Motto et al., 2017	3	1	*	*	*	**	*	6
69.			Suleiman et al., 2013	1	1, 2, 3	**	**	*	**	*	8
70.			Suleiman et al., 2018	1	1, 3	**	**	*	**	*	8
71.		Zimbabwe	Kudinhaa and Simango, 2002	3	1	*	*	*	**	*	6
72.			Perry et al., 1987	2	1, 2	**	**	*	**	*	8
73.	Asia	Bangladesh	Kayesh et al., 2014	1	1, 2, 3	**	**	*	**	*	8
74.			Islam et al., 2014	1	1	**	*	**	**	*	8
75.		China	Bi et al., 2016	3	1, 2, 3	**	**	**	**	*	9
76.			Cao et al., 2007	2	1, 2	*	*	*	**	*	6
77.			Cheng et al., 2019	2	1, 2, 3	**	**	*	**	*	8
78.			Gao et al., 2016	2	1, 2, 3	**	*	*	**	*	7
79.			Li et al., 2009	1	1	**	**	*	**	*	8
80.			Memon et al., 2012	3	1, 2	**	**	**	**	*	9
81.			Zhang et al., 2017	2	2	*	**	*	**	*	7
82.		Indonesia	Harjanti et al., 2018	1	1, 2	*	*	*	**	*	6
83.			Lucia et al., 2017	1	1, 2	*	*	*	**	*	6
84.		Iran	Atyabi et al., 2006	3	1, 2, 3	**	*	*	**	*	7
85.			Haghkhah et al., 2009	3	1, 2	*	**	*	**	*	7
86.			Haghkhah et al., 2011	1	1, 2, 3	**	**	*	**	*	8
87.			Hashemi et al., 2011	3	2, 3	**	**	*	**	*	8
88.			Jamali et al., 2014	2	1	*	*	*	**	*	6
89.			Kalantari et al., 2013	1	1, 2, 3	**	**	*	**	*	8
90.			Marashifard et al., 2019	1	3	**	*	*	**	*	7
91.			Moatamedi et al., 2007	1	2	*	*	**	**	*	7
92.			Momtaz, 2010	3	3	**	**	*	**	*	8
93.			Panahi and Saei, 2019	3	1	*	**	*	**	*	7
94.			Reza et al., 2011	3	1, 2	**	**	*	**	*	8
95.		Iraq	Abdulkadhim et al., 2012	1	1	**	**	*	**	*	8
96.			Hussein, 2012	1	1, 2, 3	**	**	*	**	*	8
97.		Jordan	Alekish, 2015	1	1, 2, 3	**	**	*	**	*	8
98.			Lafi et al., 1994	3	1, 2	**	**	*	**	*	8
99.		Malaysia	Othman and Bahaman, 2005	1	1, 2, 3	**	*	*	**	*	7
100.		Nepal	Shrestha and Bindari, 2012	1	1, 2, 3	**	**	*	**	*	8
101.		Pakistan	Ali et al., 2011	1	1, 2, 3	**	**	*	**	*	8
102.			Baloch et al., 2013	2	1	*	*	*	**	*	6
103.			Farooq et al., 2008	3	1, 2, 3	*	*	*	**	*	6
104.			Rafiullah et al., 2017	3	1, 3	*	**	*	**	*	7
105.			Umar et al., 2013	3	1, 2, 3	**	**	*	**	*	8
106.		South Korea	Nam et al., 2010	3	1, 2, 3	**	**	*	**	*	8
107.		Sri Lanka	Sanotharan et al., 2016	1	1, 2, 3	**	**	*	**	*	8
108.		Thailand	Suriyasathaporn, 2011	1	1, 2	*	**	*	**	*	7
109.		Turkey	Bal et al., 2010	1	1, 2	*	**	*	**	*	7
110.			Boynukara et al., 2008	1	1	*	**	*	**	*	7
111.			Kirkan et al., 2003	3	1	*	*	*	**	*	6
112.			Turutoglu et al., 2002	3	1, 2, 3	*	**	*	**	*	7
113.		Vietnam	Ostensson et al., 2013	1	1, 2	**	**	*	**	*	8
114.	Europe	Croatia	Macesic et al., 2012	3	1, 3	**	**	*	**	*	8
115.			Macesic et al., 2016	1	1, 2	**	**	*	**	*	8
116.		Czech Republic	Cervinkova et al., 2013	3	1, 2	**	**	**	**	*	9
117.			Vikova et al., 2017	2	1, 2	**	**	**	**	*	9
118.		Estonia	Kalmus et al., 2006	2	1, 2, 3	**	**	*	**	*	8
119.		Finland	Pyorala et al., 2011	3	1, 2, 3	*	**	*	**	*	7
120.			Vakkamaki et al., 2017	3	1, 2, 3	*	*	**	**	*	7
121.		Germany	Edinger et al., 2000	2	1, 2, 3	*	*	*	**	*	6
122.			Soltau et al., 2016	2	1, 2	**	**	*	**	*	8
123.		Italy	Bortolami et al., 2015	1	1, 2	*	*	*	**	*	6
124.			Ceniti et al., 2017	2	1, 2	*	*	*	**	*	6
125.		Kosovo	Sylejmani et al., 2016	1	1, 2, 3	**	**	*	**	*	8
126.		Lithuania	Klimiene et al., 2011	3	1, 2	**	**	*	**	*	8
127.		Netherlands	Doofer et al., 1998	2	3	**	**	**	**	*	9
128.			Miltenburg et al., 1996	2	1, 2	**	**	*	**	*	8
129.			Swinkels et al., 2013	2	1	*	*	*	**	*	6
130.		Norway	Bakken,1981	1	1	**	**	*	**	*	8
131.		Poland	Hameed et al., 2006	3	1, 2, 3	*	*	*	**	*	6
132.			Krukowski et al., 2000	3	1, 2, 3	*	**	*	**	*	7
133.			Szczubial et al., 2012	1	1, 2	*	*	*	**	*	6
134.		Serbia	Marija et al., 2016	3	1	**	*	*	**	*	7
135.			Zutic et al., 2012	1	1	**	**	*	**	*	8
136.		Slovakia	Idriss et al., 2013	2	1, 2, 3	**	*	*	**	*	7
137.		Sweden	Bengtsson et al., 2009	2	1, 2, 3	**	**	*	**	*	8
138.			Hangnestam et al., 2007	2	1, 2, 3	**	**	*	**	*	8
139.			Shitandi and Kihumbu, 2004	1	1, 2, 3	**	**	*	**	*	8
140.		Switzerland	Graber et al., 2009	1	1	**	*	*	**	*	7
141.		United Kingdom	Bradley and Green, 2001	2	3	**	*	*	**	*	7
142.			Breen et al., 2009	2	2, 3	**	**	*	**	*	8
143.			Davies et al., 2015	2	2	**	**	*	**	*	8
144.			Milne et al., 2002	2	1, 2	**	**	*	**	*	8
145.	Latin America	Argentina	Dieser et al., 2013	1	1, 2, 3	**	**	*	**	*	8
146.			Gonzalez et al., 1980	1	1	**	**	*	**	*	8
147.			Lasango et al., 2011	1	2	**	**	*	**	*	8
148.		Brazil	Budri et al., 2015	1	1	*	*	*	**	*	6
149.			Freitas et al., 2008	1	1	**	*	*	**	*	7
150.			Mesquita et al., 2018,	3	1, 2	**	**	*	**	*	8
151.			Pardo et al., 2007	3	1	**	**	*	**	*	8
152.			Silva et al., 2013	3	1	**	**	*	**	*	8
153.		Ecuador	Amer et al., 2018	3	1, 2	**	**	*	**	*	8
154.		Trinidad and Tobago	Adesiyun et al., 1998	2	1	**	**	*	**	*	8
155.	North America	Canada	Brooks et al., 1982	3	1, 2	**	**	*	**	*	8
156.			Condas et al., 2016	3	1	**	**	**	**	*	9
157.			Lago et al., 2010	2	1, 3	**	*	*	**	*	7
158.			Saini et al., 2013	3	3	**	**	*	**	*	8
159.		Mexico	Leon-Galvan et al., 2015	3	1, 2, 3	**	*	**	**	*	8
160.		United States of America	Anderson et al., 2011	3	1	**	**	*	**	*	8
161.			Erskine et al., 2002	2	1, 2, 3	**	*	*	**	*	7
162.			Ganda et al., 2016	2	1, 2, 3	*	*	**	**	*	7
163.			Gillespie et al., 2009	2	1	**	**	*	**	*	8
164.			Green et al., 2002	2	2, 3	**	**	*	**	*	8
165.			Morse et al., 1986	2	1	*	*	*	**	*	6
166.			Pankey et al., 1991	3	1, 2	**	**	*	**	*	8
167.			Sargeant et al., 1998	2	1, 2	**	**	*	**	*	8
168.			Schrick et al., 2001	1	1, 2, 3	*	*	*	**	*	6
169.			Wilson et al., 1997	3	1, 2, 3	**	**	*	**	*	8
170.	Oceania	Australia	Daniel et al., 1982	2	1, 2	*	*	*	**	*	6
171.			Phuektes et al., 2001	2	2	*	*	*	**	*	6
172.			Plozza et al., 2011	1	1, 2, 3	**	**	*	**	*	8
173.			Wanasinghe and Frost, 1979	1	1, 2	**	**	*	**	*	8
174.		New Zealand	Petrovski et al., 2009	2	1, 2	**	**	*	**	*	8

Note: SCM: subclinical mastitis; CM: clinical mastitis; M: mastitis; *S.: Staphylococcus* species; *St.: Streptococcus* species; *Ec: Escherichia coli*. # Sample representation = *: representative; **: truly representative. Size of sample = *: given; **: sample design used. Methods employed = *: cultural and biochemical tests; **: molecular methods. Prevalence value = *: calculated; **: mentioned. Assessment of outcome = *: individual assessment; **: double assessment. * Star indicates the number given to each category, i.e., * = 1; ** = 2. The reference details are provided in Supplementary File S3.

**Table 2:** Details for the univariate meta-regression analysis of moderator variables in the major mastitis pathogen prevalence studies from the world.

No.	Predictors	Model Results			Mixed-Effects Model Results				Test of Moderators	
		Estimate	Standard Error	Z Value (Test Statistic)	Tau^2^ (Estimated Residual Heterogeneity)	I^2^ (%) (Residual Heterogeneity)	H^2^ (Sampling Variability)	R^2^ (%) (Amount of Heterogeneity)	QM (Cochran Q Value for Moderators)	*p*-Value
*Staphylococcus* species										
1.	Year	−4.5307	3.7787	−1.1990	0.0558	99.65	286.24	0.24	1.7404	0.1871 ^NS^
2.	Continents	0.5320	0.2323	2.2907	0.0530	99.61	256.72	5.25	21.5992	0.0014 **
3.	Sample size	0.4544	0.0141	32.3310	0.0562	99.70	333.93	0.00	0.0028	0.9575 ^NS^
4.	Methods	0.4565	0.0149	30.3480	0.0508	99.60	249.05	9.23	31.6856	<0.0001 **
5.	Species	0.4911	0.0483	10.1651	0.0404	99.29	140.98	27.77	133.8008	<0.0001 **
6.	Host	0.4889	0.0538	9.0841	0.0560	99.72	352.49	0.00	0.4434	0.5055 ^NS^
7.	Countries	0.2778	0.0690	4.0253	0.0380	99.47	186.93	32.18	182.9442	<0.0001 **
*Streptococcus* species										
1.	Year	−5.7737	2.6655	−2.1661	0.0287	99.36	155.48	1.60	5.2140	0.0224 *
2.	Continents	0.3118	0.0172	18.1804	0.0294	99.32	147.40	0.00	3.2641	0.6593 ^NS^
3.	Sample size	0.3130	0.0108	28.9534	0.0293	99.46	185.72	0.00	0.0422	0.8372 ^NS^
4.	Methods	0.3071	0.0109	28.0433	0.0278	99.32	147.74	4.74	14.4731	0.0007 **
5.	Species	0.1201	0.1705	0.7043	0.0285	98.74	79.37	2.36	24.9063	0.1636 ^NS^
6.	Host	0.3174	0.0540	5.8828	0.0293	99.49	195.63	0.00	0.0080	0.9286 ^NS^
7.	Countries	0.2389	0.0680	3.5142	0.0258	99.26	134.96	11.41	75.6491	0.0015 **
*Escherichia coli*										
1.	Year	−7.502	7.3876	−1.0179	0.0338	98.80	83.01	0.10	1.1417	0.2853 ^NS^
2.	Continents	0.3747	0.0276	13.5921	0.0347	98.52	67.60	0.00	2.9948	0.7008 ^NS^
3.	Sample size	0.3790	0.0192	19.6934	0.0331	99.49	195.72	2.41	3.0627	0.0801 ^NS^
4.	Methods	0.3495	0.0192	18.2048	0.0298	98.75	80.19	12.01	13.3268	0.0003 **
5.	Host	0.5549	0.0765	7.2487	0.0322	99.54	215.90	4.95	5.9864	0.0144 *
6.	Countries	0.4664	0.0929	5.0216	0.0283	97.10	34.45	16.33	50.2262	0.0212 *

Note: ^NS^: not significant; *: significant (*p* < 0.05); **: highly significant (*p* < 0.01).

**Table 3:** *Staphylococcus* (*S.*) *species* prevalence estimates in the world based on various subgroup meta-analyses.

No.	Categories	Period	Number of Studies	Total Samples	Pooled Estimates		Tests of Heterogeneity				
					Prevalence (%) [CI at 95% Level)	PI (%) at 95% Level	I^2^ Value (%)	Tau Square Value	H Value	Degrees of Freedom	Cochran Q Value
1.	World	1979–2019	156	283,685	28 (24–31)	4–77	99.6	1.165	14.98	155	38,800.6 **
Year-wise											
1.	World-I	1979–2000	16	117,197	23 (12–24)	1–92	99.8	2.797	24.30	15	2432.7 **
2.	World-II	2001–2010	37	14,592	25 (18–32)	3–77	98.7	1.296	8.79	36	1813.9 **
3.	World-III	2011–2019	103	151,896	30 (26–34)	6–73	98.8	0.852	9.28	102	8720.9 **
Continent-wise											
1.	Africa	1987–2019	67	13,702	32 (27–37)	6–76	97.3	0.934	6.06	66	2701.3 **
2.	Asia	1994–2019	36	13,396	27 (21–34)	5–74	98.2	0.964	7.49	35	1578.1 **
3.	Europe	1981–2017	27	131,152	23 (17–29)	4–67	99.2	0.836	11.12	26	4228.9 **
4.	Latin America	1980–2018	9	2587	51 (27–75)	2–98	98.9	2.491	9.69	8	805.5 **
5.	North America	1982–2016	13	116,927	13 (8–20)	2–57	99.5	0.876	13.79	12	568.0 **
6.	Oceania	1979–2011	4	5921	31 (16–50)	1–96	98.6	0.657	8.54	3	151.9 **
Period-wise											
1.	Africa-I	1987–2010	14	3108	24 (12–43)	1–92	98.6	2.588	8.42	13	856.6 **
2.	Africa-II	2011–2019	53	10,594	34 (30–38)	11–68	95.3	0.501	4.63	52	1018.6 **
3.	Asia-I	1994–2010	12	5410	28 (21–36)	8–62	96.4	0.395	5.26	11	192.7 **
4.	Asia-II	2011–2019	24	7986	27 (19–37)	3–80	98.2	1.282	7.50	23	1269.7 **
5.	Europe-I	1981–2009	11	6438	23 (14–36)	2–78	98.9	1.115	9.41	10	708.0 **
6.	Europe-II	2011–2017	16	124,714	22 (16–30)	5–63	98.9	0.655	9.58	15	2219.0 **
7.	Latin America-I	1980–2008	4	615	62 (19–92)	0–100	98.5	3.959	8.11	3	373.8 **
8.	Latin America-II	2013–2018	5	1972	43 (22–66)	2–97	98.3	1.143	7.69	4	369.1 **
9.	North America-I	1982–2009	9	110,401	12 (7–21)	1–59	99.3	0.857	11.82	8	487.5 **
10.	North America-II	2011–2016	4	6526	16 (7–32)	0–95	98.6	0.887	8.46	3	70.1 **
11.	Oceania-I	1979–2009	3	5817	23 (14–35)	0–100	97.5	0.266	6.27	2	74.7 **
12.	Oceania-II	2011	1	104	60	-	-	-	-	-	-
Host species-wise											
1.	Cattle	1979–2019	147	281,390	28 (24–31)	4–76	99.6	1.163	15.27	146	38,517.2 **
2.	Buffalo	2008–2019	13	2255	31 (20–46)	4–84	96.9	1.131	5.71	12	291.7 **
Method-wise											
1.	Cultural Examination	1979–2019	132	154,085	27 (23–31)	4–77	99.1	1.210	10.70	131	11,559.6 **
2.	Molecular methods	2008–2019	19	124,004	35 (26–45)	7–80	99.0	0.874	10.11	18	1769.9 **
3.	Other methods	1980–2019	13	7989	22 (16–29)	5–58	96.6	0.478	5.39	12	363.4 **
Bacterial species-wise											
1.	*S. aureus*	1979–2019	137	273,336	25 (21–29)	3–76	99.5	1.287	14.62	136	15,898.9 **
2.	CNS	1996–2017	18	123,118	20 (14–28)	3–65	98.8	0.842	8.97	17	2022.0 **
3.	*S. auricularis*	2010	1	100	14	-	-	-	-	-	-
4.	*S. captis*	2010 and 2019	2	268	7 (4–10)	-	0.0	0.0	1.00	1	0.13 ^NS^
5.	*S. caseolyticus*	2002	1	131	1.5	-	-	-	-	-	-
6.	*S. chromogenes*	2002–2019	9	6453	11 (6–19)	1–56	97.1	0.866	5.82	8	170.7 **
7.	*S. cohnii*	2010 and 2015	2	136	4 (2–9)	-	0.0	0.0	1.00	1	0.45 ^NS^
8.	*S. devriesei*	2019	1	168	1.6	-	-	-	-	-	-
9.	*S. epidermidis*	1980–2019	27	11,337	9 (6–13)	1–46	95.8	1.005	4.89	26	1047.1 **
10.	*S. haemolyticus*	2010–2019	3	5417	8 (3–23)	0–100	96.5	1.047	5.32	2	77.4 **
11.	*S. hominis*										
12.	*S. hyicus*	2002–2016	8	1991	6 (3–12)	1–39	93.0	0.732	3.78	7	65.1 **
13.	*S. intermidius*	2004–2015	8	2808	4 (2–8)	1–26	85.8	0.599	2.65	7	52.3 **
14.	*S. kloosii*	2002 and 2019	2	299	1 (0–3)	-	0.0	0.0	1.00	1	0.03 ^NS^
15.	*S. lentus*	2002 and 2011	3	254	4 (2–7)	0–71	0.0	0.0	1.00	2	0.98 ^NS^
16.	*S. lugdunensis*	2019	1	168	0.6	-	-	-	-	-	-
17.	*S. muscae*	2019	1	131	0.8	-	-	-	-	-	-
18.	*S. pasteuri*	2019	1	168	2.4	-	-	-	-	-	-
19.	*S. saprophyticus*	2002–2019	5	1314	3 (1–9)	0–72	86.3	1.586	2.70	4	39.6 **
20.	*S. sciuri*	2002–2019	4	608	4 (1–14)	0–94	90.8	1.453	3.29	3	45.6 **
21.	*S. simulans*	2002–2018	7	6083	5 (2–10)	0–41	92.5	0.882	3.65	6	55.1 **
22.	*S. warneri*	2010–2019	4	383	2 (1–7)	0–71	56.9	0.826	1.52	3	11.65 *
23.	*S. xylosus*	2002–2019	7	5896	5 (3–10)	1–30	87.0	0.505	2.77	6	71.3 **
24.	*S. species*	1991–2018	34	119,139	25 (18–35)	2–83	99.5	1.651	14.13	33	3280.5 **
Type of mastitis											
1.	Subclinical mastitis	1979–2019	50	15,165	30 (24–36)	4–79	98.3	1.176	7.69	49	20.2.3 **
2.	Clinical mastitis	1982–2019	27	17,344	22 (15–32)	2–84	99.3	1.878	12.15	26	2115.3 **
3.	Mastitis	1982–2019	79	251,176	29 (25–33)	6–73	99.6	0.882	16.07	78	33,759.1 **

Note: CI: confidence interval; PI: prediction interval; CNS: coagulase-negative *Staphylococcus*; ^NS^: not significant; *: significant (*p* < 0.05), **: highly significant (*p* < 0.01), ‘-‘meaning values not obtained due to single study was available.

**Table 4:** *Streptococcus* (*St.*) *species* prevalence estimates in the world based on various subgroup meta-analyses.

No.	Categories	Period		Number of Studies	Total Samples	Pooled Estimates		Tests of Heterogeneity				
						Prevalence (%) [CI at 95% Level]	PI (%) at 95% Level	I^2^ Value (%)	Tau Square Value	H Value	Degrees of Freedom	Cochran Q Value
1.	World	1979–2019		129	272,539	12 (10–14)	2–54	99.3	1.151	11.96	128	10,173.8 **
			Year-wise									
1.	World-I	1979–2000		13	116,643	7 (5–11)	1–34	99.2	0.661	10.85	12	463.9 **
2.	World-II	2001–2010		34	13,505	11 (8–16)	1–55	98.2	1.213	7.47	33	1571.2 **
3.	World-III	2011–2019		82	142,391	14 (11–17)	2–57	98.4	1.114	8.00	81	6570.4 **
			Continent-wise									
1.	Africa	1987–2019		56	11,217	11 (9–13)	2–41	93.9	0.768	4.04	55	876.7 **
2.	Asia	1994–2019		31	12,993	14 (9–21)	1–71	98.6	1.656	8.35	30	3048.0 **
3.	Europe	1996–2017		23	130,131	12 (8–18)	1–62	99.4	1.359	13.12	22	3034.1 **
4.	Latin America	1980–2018		5	2147	15 (5–36)	0–95	98.3	1.794	7.69	4	587.8 **
5.	North America	1982–2016		9	109,992	9 (8–11)	5–16	85.3	0.056	2.61	8	64.1 **
6.	Oceania	1979–2011		5	6059	25 (12–44)	1–91	98.6	0.929	8.39	4	199.1 **
			Period-wise									
1.	Africa-I	1987–2010		15	3247	8 (5–14)	1–56	95.4	1.398	4.65	14	315.3 **
2.	Africa-II	2011–2019		41	7970	12 (9–14)	3–37	91.9	0.527	3.52	40	493.1 **
3.	Asia-I	1994–2010		10	4655	11 (6–19)	1–62	96.8	1.159	5.63	9	282.1 **
4.	Asia-II	2011–2019		21	8338	17 (10–27)	1–78	98.5	1.780	8.03	20	2735.8 **
5.	Europe-I	1996–2009		10	6782	9 (5–17)	1–59	98.8	1.213	9.22	9	847.9 **
6.	Europe-II	2011–2017		13	123,349	15 (8–25)	1–71	99.2	1.340	10.93	12	1548.5 **
7.	Latin America-I	1980		1	100	12	-	-	-	-	-	-
8.	Latin America-II	2011–2018		4	2047	16 (4–44)	0–100	98.9	2.207	9.33	3	587.0 **
9.	North America-I	1982–2002		7	109,409	9 (7–11)	5–17	89.0	0.067	3.02	6	57.6 **
10.	North America-II	2015 and 2016		2	583	10 (8–13)	-	0.0	0.0	1.00	1	0.11 ^NS^
11.	Oceania-I	1982–2009		4	5955	23 (10–46)	0–98	99.0	1.113	9.90	3	180.2 **
12.	Oceania-II	2011		1	104	34	-	-	-	-	-	-
			Host species-wise									
1.	Cattle	1979–2019		123	271,307	12 (10–15)	2–55	99.3	1.175	12.33	122	10,122.8 **
2.	Buffalo	2008–2017		7	1232	10 (6–17)	2–45	85.8	0.501	2.66	6	49.2 **
			Method-wise									
1.	Cultural Examination	1979–2019		119	150,988	12 (10–14)	2–49	98.5	0.939	8.13	118	6571.5 **
2.	Molecular methods	2007–2018		10	121,977	17 (8–33)	1–87	99.4	2.060	12.90	9	3525.2 **
3.	Other methods	1980 and 1994		2	347	3 (0–21)	-	86.2	1.874	2.69	1	20.7 **
			Bacterial species-wise									
1.	*St. acidominimus*	2010 and 2013		2	1151	2 (1–3)	-	0.0	0.0	1.00	1	4.47 *
2.	*St. agalactiae*	1979–2018		83	138,904	9 (7–12)	1–57	99.0	1.662	10.07	82	6296.6 **
3.	*St. anginosus*	1994 and 2010		2	281	1 (0–3)	-	0.0	0.0	1.00	1	1.73 ^NS^
4.	*St. bovis*	2010 and 2011		4	321	3 (1–5)	1–11	0.0	0.0	1.00	3	1.66 ^NS^
5.	*St. constellatus*	2010		1	34	3	-	-	-	-	-	-
6.	*St. dysgalactiae*	1980–2018		55	141,844	6 (5–8)	1–36	98.5	1.152	8.28	54	3131.1 **
7.	*St. equinus*	2001–2011		4	1571	2 (1–5)	0–40	75.4	0.436	2.02	3	23.8 **
8.	*St. equisimilis*	2017		1	65	3	-	-	-	-	-	-
9.	*St. faecalis*	2002–2017		4	385	4 (2–8)	0–46	50.2	0.339	1.42	3	8.15 *
10.	*St. intermedius*	2017		2	319	6 (4–10)	-	0.0	0.0	1.00	1	0.27 ^NS^
11.	*St. mitis*	2011 and 2018		2	104	2 (0–7)	-	0.0	0.0	1.00	1	1.45 ^NS^
12.	*St. plurianimalium*	2017		1	65	1.5	-	-	-	-	-	-
13.	*St. pneumonia*	2009		1	71	1.4	-	-	-	-	-	-
14.	*St. pyogenes*	2014		1	47	10.6	-	-	-	-	-	-
15.	*St. salivaris*	2010		1	34	2	-	-	-	-	-	-
16.	*St. sanguinis*	2011–2018		2	104	8 (0–63)	-	86.6	4.151	2.73	1	20.1 **
17.	*St. uberis*	1980–2018		52	139,050	9 (7–12)	1–53	99.0	1.429	9.96	51	4000.7 **
18.	*St. zooepidemicus*	2010		1	130	3.9	-	-	-	-	-	-
19.	*St. species*	1982–2019		48	120,504	10 (8–12)	2–37	97.5	0.687	6.30	47	959.9 **
Type of mastitis												
1.	Subclinical mastitis	1979–2019		40	12,853	13 (10–16)	3–44	95.1	0.691	4.52	39	543.7 **
2.	Clinical mastitis	1982–2019		26	16,848	13 (8–20)	1–72	99.2	1.811	11.13	25	2556.0 **
3.	Mastitis	1982–2018		63	242,838	11 (9–15)	1–53	99.5	1.138	14.49	62	5973.1 **

Note: CI: confidence interval; PI: prediction interval; ^NS^: not significant; *: significant (*p* < 0.05); **: highly significant (*p* < 0.01).

**Table 5:** *Escherichia coli* prevalence estimates in the world based on various subgroup meta-analyses.

No.	Categories	Period	Number of Studies	Total Samples	Pooled Estimates		Tests of Heterogeneity				
					Prevalence (%) [CI at 95% Level]	PI (%) at 95% Level	I^2^ Value (%)	Tau Square Value	H Value	Degrees of Freedom	Cochran Q Value
1.	World	1996–2019	92	257,473	11 (9–13)	1–50	98.9	1.154	9.68	91	17,511.1 **
Year-wise											
1.	World-I	1996–2000	5	113,108	5 (1–20)	0–95	99.8	2.685	24.61	4	10,139.0 **
2.	World-II	2001–2010	23	8918	11 (7–16)	1–52	97.7	1.088	6.59	22	540.3 **
3.	World-III	2011–2019	64	135,447	11 (40–47)	12–80	98.2	0.962	7.39	63	3439.4 **
Continent-wise											
1.	Africa	2006–2018	45	8142	10 (8–14)	1–50	95.6	1.093	4.77	44	792.0 **
2.	Asia	2002–2019	21	11,561	12 (9–17)	3–42	97.0	0.579	5.80	20	394.7 **
3.	Europe	1996–2017	16	129,580	10 (6–15)	1–53	99.6	1.128	15.67	15	5117.3 **
4.	Latin America	2013	1	1117	2	-	-	-	-	-	-
5.	North America	1997–2016	8	106,969	11 (4–27)	0–87	99.1	2.396	10.74	7	1399.0 **
6.	Oceania	2011	1	104	28	-	-	-	-	-	-
Period-wise											
1.	Africa-I	2006–2010	6	636	5 (2–12)	0–49	83.1	0.860	2.43	5	30.1 **
2.	Africa-II	2011–2018	39	7506	11 (8–15)	2–51	95.8	1.038	4.88	38	714.9 **
3.	Asia-I	2002–2010	6	4309	9 (6–12)	2–27	87.6	0.199	2.85	5	28.6 **
4.	Asia-II	2011–2019	15	7252	15 (10–21)	3–48	96.8	0.578	5.63	15	249.9 **
5.	Europe-I	1996–2009	11	11,089	12 (6–20)	1–61	99.1	1.103	10.61	10	679.6 **
6.	Europe-II	2011–2017	5	118,491	6 (3–14)	0–62	97.0	0.810	5.73	4	75.2 **
8.	Latin America-II	2013	1	1117	2	-	-	-	-	-	-
9.	North America-I	1997–2010	5	105,992	12 (3–44)	0–99	99.5	3.742	14.16	4	1062.1 **
10.	North America-II	2013–2016	3	977	9 (8–11)	2–29	0.0	0.0	1.00	2	0.09 ^NS^
12.	Oceania-II	2011	1	104	28	-	-	-	-	-	-
Host species-wise											
1.	Cattle	1996–2019	89	256,295	10 (8–13)	1–51	99.0	1.187	9.82	88	16,809.4 **
2.	Buffalo	2011–2018	6	1178	25 (9–52)	0–96	97.9	2.070	6.84	5	167.2 **
Method-wise											
1.	Cultural Examination	1996–2019	87	131,774	10 (8–12)	1–47	97.5	1.056	6.29	86	10,463.3 **
2.	Molecular methods	1998–2018	8	125,838	23 (11–42)	1–89	99.8	1.662	25.41	7	5387.8 **
Type of mastitis											
1.	Subclinical mastitis	2001–2019	29	6125	10 (7–14)	1–47	94.6	1.002	4.29	28	671.7 **
2.	Clinical mastitis	1996–2019	16	15,337	19 (14–25)	5–52	98.4	0.494	7.79	15	687.8 **
3.	Mastitis	1997–2018	47	236,011	9 (6–12)	1–50	98.9	1.312	9.59	46	8384.4 **

Note: CI: confidence interval; PI: prediction interval; ^NS^: not significant; **: highly significant (*p* < 0.01).

## Data Availability

The data presented in this study are available on request from the first author.

## Supplementary Materials

The following are available online at https://www.mdpi.com/article/10.3390/pathogens10050545/s1, The supplementary materials for this article can be found online. Supplementary File S1: PRISMA checklist for the systematic review and meta-analysis. Supplementary File S2: Tables of the manuscript. Supplementary File S3: Reference list of the studies included for meta-analysis from the world.
