# Supplementary material for: An Understanding of the Global Status of Major Bacterial Pathogens of Milk Concerning Bovine Mastitis: A Systematic Review and Meta-Analysis (Scientometrics)
Source: Pathogens. 2021 Apr 30;10(5):545. doi: 10.3390/pathogens10050545 (PMC8147236; doi:10.3390/pathogens10050545)
Supplement: Supplementary file 1 [file pathogens-10-00545-s001.zip › Supplementary files/Supplementary file S2 tables for MMP manuscript.pdf]

Table S1. Particulars of major mastitis pathogens (MMP) prevalence studies from India with quality assessment scores included for a meta-analysis

No.	Zone	State name	Studies [Author and Year]	SCM-1, CM-2, M-3	*S.* -1, *St.*-2, *Ec*-3	Quality assessment of the studies#					
						Sample representation (Maximum score=2)	Size of sample (Maximum score=2)	Methods employed (Maximum score=2)	Prevalence values (Maximum score=2)	Assessment of outcome (Maximum score=2)	Total score (Maximum score=10)
1.			Bhanot et al., 2012	3	1, 2, 3	*	*	*	**	*	6
2.			Bulla et al., 2006	1	1, 2	**	**	*	**	*	8
3.			Charaya et al., 2013	1	1, 2	**	**	*	**	*	8
4.			Charaya et al., 2014	2	1, 2, 3	*	*	*	**	*	6
5.			Guha and Gera, 2011	1	1, 2, 3	**	**	*	**	*	8
6.			Guha et al., 2012	1	1, 2, 3	**	**	*	**	*	8
7.			Pankaj et al., 2012	1	1, 2	**	**	*	**	*	8
8.			Pankaj et al., 2013	1	1, 2	**	**	*	**	*	8
9.		Haryana	Sharma and Sindhu, 2007	3	1, 2, 3	**	*	*	**	*	7
10.			Sharma et al., 2006	1	1, 2	**	**	*	**	*	8
11.			Sharma et al., 2012	1	1, 2	*	*	*	**	*	6
12.			Sharma et al., 2018	3	1, 2, 3	**	**	*	**	*	8
13.			Sindhu et al., 2009	1	1, 2	**	**	*	**	*	8
14.			Sindhu et al., 2012	1	1	**	**	*	**	*	8
15.			Singh and Garg, 2011	1	1, 2, 3	*	*	*	**	*	6
16.	North		Thakur et al., 2018	3	1, 2	*	*	*	**	*	6
17.			Bhat et al., 2017	2	1, 2, 3	*	*	*	**	*	6
18.			Dar et al., 2014	1	1, 2, 3	**	**	*	**	*	8
19.		Jammu and Kashmir	Ganai et al., 2015	3	3	*	**	*	**	*	7
20.			Qadri et al., 2017	1	1, 2, 3	*	*	*	**	*	6
21.			Sudhan et al., 2005	1	1, 3	**	*	*	**	*	7
22.			Tufani et al., 2012	3	1, 3	**	*	*	**	*	7
23.			Bansal et al., 2015	3	1	*	*	*	**	*	6
24.			Kour et al., 2017	3	1, 2, 3	*	*	**	**	*	7
25.		Punjab	Mir et al., 2014	1	1, 2	**	**	*	**	*	8
26.			Prabhakar et al., 1995	2	1, 2, 3	**	*	*	**	*	7
27.			Singh et al., 2018	3	1, 2, 3	*	*	**	**	*	7
28.			Ali et al., 2015	1	1, 2, 3	*	*	*	**	*	6
29.		Uttar Pradesh	Kumar et al., 2010	2	1, 3	**	**	*	**	*	8
30.			Sharma et al., 2015	3	1	**	*	*	**	*	7
31.			Verma et al., 2017	3	1, 2, 3	*	*	*	**	*	6
32.		Uttarakhand	Tripathi et al., 2018	2	1, 2, 3	*	*	*	**	*	6
33.		Assam	Gogoi et al., 2017	1	1, 3	**	**	*	**	*	8
34.			Sharma and Brinty, 2014	3	1	**	**	*	**	*	8
35.			Kumar et al., 2015	3	1, 2, 3	*	*	*	**	*	6
36.	East	Jharkhand	Patnaik et al., 2014	1	1, 2, 3	**	**	*	**	*	8
37.			Ranjan et al., 2011	3	1, 2, 3	*	*	*	**	*	6
38.		Mizoram	Das et al., 2015	1	1	*	*	*	**	*	6
39.		Odisha	Mohanty et al., 2013	3	1, 2, 3	*	*	*	**	*	6

40.		Sikkim	Dubal et al., 2010	3	1, 2, 3	**	**	*	**	*	8
41.		West Bengal	Paul et al., 2013	1	1, 2	*	*	*	**	*	6
42.	West	Gujarat	Patel et al., 2012	1	1, 2, 3	**	**	*	**	*	8
43.			Singh et al., 2016	3	1, 2, 3	**	**	*	**	*	8
44.			Thakor et al., 2013	1	1, 2	**	**	*	**	*	8
45.		Maharashtra	Bhikane et al., 2010	1	1, 2, 3	*	*	*	**	*	6
46.			Sudhakar et al., 2009	2	1, 3	*	*	*	**	*	6
47.		Rajasthan	Gupta et al., 2015	2	1, 2, 3	*	*	*	**	*	6
48.			Jena et al., 2015	1	1, 2, 3	**	**	*	**	*	8
49.			Joshi et al., 2013	1	1, 2, 3	**	*	*	**	*	7
50.			Sharma et al., 2010	1	1, 2, 3	**	**	*	**	*	8
51.			Swarnakar et al., 2017	2	1	*	*	*	**	*	6
52.	South	Andhra Pradesh	Kavitha et al., 2009	3	1, 2, 3	**	*	*	**	*	7
53.			Sudhanthiramani et al., 2015	3	1, 2, 3	**	**	*	**	*	8
54.		Karnataka	Harini and Sumathi, 2011	1	1, 2, 3	**	**	*	**	*	8
55.			Hegde et al., 2013	1	1, 2, 3	**	**	*	**	*	8
56.			Kaliwal et al., 2011	3	1	**	**	*	**	*	8
57.			Karabasanavar et al., 2019	1	1	**	**	*	**	*	8
58.			Krishnaveni et al., 2014	3	1, 2, 3	*	*	*	**	*	6
59.			Kurjogi and Kaliwal, 2011	3	1, 2, 3	**	**	*	**	*	8
60.			Prabhu et al., 2015	1	1	**	**	*	**	*	8
61.			Preethirani et al., 2015	1	1, 2, 3	**	*	**	**	*	8
62.			Sadashiv and Kaliwal, 2012	3	3	**	**	*	**	*	8
63.			Shome et al., 2011	3	1, 2, 3	**	*	**	**	*	8
64.			Swamy and Krishnamurthy,	3	1	**	**	*	**	*	8
65.		Kerala	Lakshmi and Jayavardhanan,	3	1, 2, 3	*	*	*	**	*	6
66.		Tamil Nadu	Ayyappadas and Renugadevi,	1	1, 2, 3	**	**	**	**	*	9
67.			Bharathy et al., 2015	3	1	**	**	**	**	*	9
68.			Elango et al., 2010	1	1, 2, 3	**	**	*	**	*	8
69.			Jeykumar et al., 2013	3	1, 2, 3	*	*	*	**	*	6
70.			Mubarack et al., 2012	1	1, 2, 3	*	*	*	**	*	6
71.			Navaneethan et al., 2017	2	3	*	*	**	**	*	7
72.			Saravanajayam et al., 2015	2	1, 2, 3	*	*	*	**	*	6
73.			Srinivasan et al., 2013	1	1, 2, 3	**	**	*	**	*	8
74.	Central	Madhya Pradesh	Das and Joseph, 2005	2	1, 2, 3	*	*	*	**	*	6
75.		Chhattisgarh	Sharma et al., 2007	1	1, 2, 3	**	**	*	**	*	8

Note: SCM-Subclinical mastitis, CM-Clinical mastitis, M-Mastitis, *S.-Staphylococcus species, St.-Streptococcus species, Ec-Escherichia coli.* #Sample representation=*-Representative, **-Truly representative; Size of sample=*-Given, **-Sample design used; Methods employed=*-Cultural and biochemical tests, **-Molecular methods; Prevalence value=*-Calculated, **-Mentioned; Assessment of outcome=*-Individual assessment, **-Double assessment. *-Star indicates the number given to each category, i.e., *=1, **=2. The reference details are provided in Supplementary file 3.

Table S2. Details for the univariate meta-regression analysis of moderator variables in the major mastitis pathogens prevalence studies from India

No.	Predictors	Model Results			Mixed-Effects model results				Test of Moderators	
		Estimate	Standard error	Z Value (Test Statistic)	Tau^2 (Estimated residual heterogeneity)	I^2 [%] (Residual heterogeneity)	H^2 (Sampling variability)	R^2 [%] (Amount of heterogeneity)	QM (Cochran Q value for Moderators)	P-value

Staphylococcus species										
1.	Year	-2.8177	9.5249	-0.2958	0.0413	95.90	24.37	0.00	0.1315	0.7169 NS
2.	State-wise	0.4624	0.1204	3.8395	0.0400	95.99	24.95	2.23	21.0981	0.3314 NS
3.	Sample size	0.6327	0.0224	28.2763	0.0413	95.74	23.46	0.00	0.1310	0.7174 NS
4.	Methods	0.6472	0.0223	28.9991	0.0405	96.16	26.01	0.95	1.7274	0.1887 NS
5.	Species	0.5264	0.0648	8.1227	0.0274	94.26	17.42	33.01	54.4842	<0.0001 **
6.	Host	0.6511	0.0465	13.9936	0.0413	95.91	24.46	0.00	0.1364	0.7118 NS
Streptococcus species										
1.	Year	-12.9059	7.6991	-1.6763	0.0232	93.03	14.35	2.79	2.9855	0.0840 NS
2.	State-wise	0.4277	0.1218	3.5104	0.0261	94.53	18.30	0.00	10.9322	0.8601 NS
3.	Sample size	0.3807	0.0199	19.1069	0.0224	92.79	13.87	5.91	4.8518	0.0276 *
4.	Methods	0.3950	0.0202	19.5974	0.0242	94.15	17.11	0.00	0.0743	0.7852 NS
5.	Species	0.4521	0.0215	21.0693	0.0167	91.70	12.05	29.95	32.4104	<0.0001 **
6.	Host	0.4070	0.0386	10.5460	0.0241	93.43	15.22	0.00	0.0922	0.7614 NS
Escherichia coli										
1.	Year	-13.5047	10.2458	-1.3181	0.0174	91.78	12.17	0.76	1.8458	0.1743 NS
2.	State-wise	0.3414	0.1114	3.0650	0.0212	94.49	18.15	0.00	9.8879	0.9082 NS
3.	Sample size	0.4235	0.0213	19.9266	0.0176	92.29	12.97	0.00	0.9860	0.3207 NS
4.	Methods	0.4065	0.0210	19.3205	0.0174	93.22	14.75	0.77	1.2533	0.2629 NS
5.	Host	0.3481	0.0510	6.8271	0.0172	92.04	12.57	2.20	2.0285	0.1544 NS

Note: NS-Not significant, *- Significant (P<0.05), **- Highly significant (P<0.01).

Table S3. *Staphylococcus (S.) species* prevalence estimates in India based on various subgroup meta-analyses

No.	Categories	Period	Number of studies	Total samples	Pooled estimates		Tests of Heterogeneity				
					Prevalence (%) [CI at 95% level]	PI (%) at 95% level	I^2 Value (%)	Tau square value	H value	Degrees of Freedom	Cochran Q value
1.	India	1995-2019	72	14,011	41 [38-45]	16-72	94.3	0.416	4.19	71	1014.8**
2a.	India-Period I	1995-2010	16	4,849	43 [36-51]	18-72	91.6	0.303	3.46	15	120.1**
2b.	India-Period II	2011-2019	56	9,162	41 [36-45]	15-73	93.6	0.445	3.96	55	886.6**
3.	North zone	1995-2018	31	9,144	42 [37-48]	17-72	95.3	0.378	4.60	30	500.4**
3a.	Haryana	2006-2018	16	7,593	45 [39-51]	24-69	94.1	0.194	4.11	15	189.4**
3b.	Jammu and Kashmir	2005-2017	5	602	34 [16-59]	1-96	94.5	1.230	4.26	4	134.7**
3c.	Punjab	1995-2018	5	518	35 [27-44]	13-66	73.7	0.127	1.95	4	20.6**
3d.	Uttarakhand	2018	1	142	67	-	-	-	-	-	-
3e.	Uttar Pradesh	2010-2018	4	289	38 [33-44]	27-51	0.0	0.0	1.00	3	5.1NS
4.	East zone	2010-2017	9	1,133	36 [26-49]	8-79	93.2	0.549	3.82	8	109.5**
4a.	Assam	2014 & 2017	2	138	30 [23-38]	-	0.0	0.0	1.00	1	0.04NS
4b.	Jharkhand	2011-2015	3	440	21 [16-27]	1-90	50.6	0.045	1.42	2	6.21*
4c.	Mizoram	2015	1	75	67	-	-	-	-	-	-
4d.	Odisha	2013	1	144	47	-	-	-	-	-	-
4e.	Sikkim	2010	1	276	42	-	-	-	-	-	-
4f.	West Bengal	2013	1	60	63	-	-	-	-	-	-

5.	West zone	2009-2017	10	1,009	40 [31-50]	14-73	87.5	0.336	2.83	9	78.7**
5a.	Gujarat	2012-2016	3	351	33 [28-38]	10-67	0.0	0.0	1.00	2	1.7^{NS}
5b.	Maharashtra	2009 & 2010	2	141	46 [17-78]	-	91.9	0.983	3.52	1	26.9**
5c.	Rajasthan	2010-2017	5	517	41 [29-54]	9-82	86.4	0.288	2.71	4	46.0**
6.	South zone	1998-2019	20	2,529	43 [36-51]	15-76	92.4	0.440	3.62	19	267.6**
6a.	Andhra Pradesh	2009 & 2015	2	140	22 [16-30]	-	0.0	0.0	1.00	1	0.3^{NS}
6b.	Karnataka	1998-2019	10	1,742	46 [36-56]	16-80	93.7	0.393	3.99	9	181.2**
6c.	Kerala	2016	1	31	35	-	-	-	-	-	-
6d.	Tamil Nadu	2010-2015	7	616	46 [35-59]	14-82	86.9	0.362	2.76	6	41.1**
7.	Central zone	2005 & 2007	2	196	40 [24-59]	-	84.5	0.258	2.54	1	13.4**
7a.	Chhattisgarh	2007	1	110	54	-	-	-	-	-	-
7b.	Madhya Pradesh	2005	1	86	28	-	-	-	-	-	-
8a.	Cattle	1998-2019	59	10,496	40 [35-44]	14-72	94.1	0.465	4.13	58	826.2**
8b.	Buffaloes	1995-2019	14	3,475	50 [45-55]	35-65	66.6	0.070	1.73	13	33.7**
9a.	Cultural & biochemical tests	1995-2019	66	13,012	41 [37-45]	16-72	94.2	0.408	4.14	65	915.6**
9b.	Molecular methods	2010-2018	7	1,019	47 [35-60]	12-85	93.3	0.439	3.85	6	93.5**
10a.	*Staphylococcus aureus*	1995-2018	39	4,396	38 [33-44]	13-71	91.5	0.459	3.44	38	423.4**
10b.	*CNS*	1995-2015	7	1,242	24 [15-36]	4-70	94.1	0.526	4.11	6	164.7**
10c.	*S. chromogenes*	2011	1	115	38	-	-	-	-	-	-
10d.	*S. epidermidis*	2005-2018	9	830	24 [15-36]	3-73	91.5	0.749	3.43	8	130.7**
10e.	*S. haemolyticus*	2011	1	115	10	-	-	-	-	-	-
10f.	*S. hyicus*	2012 &2013	2	194	3 [1-6]	-	0.0	0.0	1.00	1	0.77^{NS}
10g.	*S. intermedius*	2012	1	150	2	-	-	-	-	-	-
10h.	*S. sciuri*	2011	1	115	6	-	-	-	-	-	-
10i.	*S. simulans*	2011	1	115	0.9	-	-	-	-	-	-
10j.	*S. species*	2005-2019	40	10,062	41 [36-46]	14-74	95.7	0.473	4.80	39	682.3**
11a.	Subclinical mastitis	2005-2019	36	3,988	46 [40-51]	18-76	90.8	0.422	3.30	35	532.9**
11b.	Clinical mastitis	1995-2018	10	1,186	34 [26-43]	11-68	88.7	0.338	2.97	9	93.2**
11c.	Mastitis	1998-2018	26	8,837	39 [33-44]	15-69	95.6	0.349	4.74	25	370.9**

Note: CI-Confidence Interval, PI-Prediction Interval, CNS-Coagulase Negative Staphylococcus, NS-Not significant, *-Significant ($P<0.05$), **- Highly Significant ($P<0.01$).

Table S4. *Streptococcus (St.) species* prevalence estimates in India based on various subgroup meta-analyses

No.	Categories	Period	Number of studies	Total samples	Pooled estimates		Tests of Heterogeneity				
					Prevalence (%) [CI at 95% level]	PI (%) at 95% level	I^2 Value (%)	Tau square value	H value	Degrees of Freedom	Cochran Q value
1.	India	1995-2018	57	12,314	18 [16-21]	5-48	94.1	0.474	4.10	56	827.9**
2a.	India-Period I	1995-2010	13	4,543	18 [12-26]	4-55	92.5	0.567	3.66	12	207.2**
2b.	India-Period II	2011-2018	44	7,771	18 [15-22]	5-47	92.0	0.445	3.53	43	609.6**
3.	North zone	1995-2018	26	8,664	23 [19-27]	9-46	92.8	0.251	3.73	25	255.6**
3a.	Haryana	2006-2018	15	7,443	28 [25-32]	17-43	85.5	0.078	2.63	14	73.4**
3b.	Jammu and Kashmir	2014-2017	3	481	12 [10-16]	2-45	0.0	0.0	1.00	2	0.09^{NS}
3c.	Punjab	1995-2018	4	389	22 [14-31]	4-67	71.0	0.160	1.86	3	14.7**
3d.	Uttarakhand	2018	1	142	23	-	-	-	-	-	-
3e.	Uttar Pradesh	2010-2018	3	209	13 [4-34]	0-100	82.5	0.896	2.39	2	26.7**
4.	East zone	2010-2015	6	920	11 [6-20]	1-61	90.4	0.719	3.23	5	80.7**

4a.	Jharkhand	2011-2015	3	440	9 [5-15]	0-99	68.0	0.181	1.77	2	9.24**
4b.	Odisha	2013	1	144	32	-	-	-	-	-	-
4c.	Sikkim	2010	1	276	3	-	-	-	-	-	-
4d.	West Bengal	2013	1	60	17	-	-	-	-	-	-
5.	West zone	2010-2016	8	727	16 [10-26]	3-59	87.2	0.573	2.80	7	47.7**
5a.	Gujarat	2012-2016	3	351	25 [21-30]	0-98	62.5	0.098	1.63	2	8.85^{NS}
5b.	Maharashtra	2010	1	41	10	-	-	-	-	-	-
5c.	Rajasthan	2010-2015	4	335	11 [5-25]	0-89	85.8	0.721	2.65	3	24.6**
6.	South zone	2009-2016	15	1,807	16 [12-22]	4-46	87.4	0.440	2.82	14	122.1**
6a.	Andhra Pradesh	2009 & 2015	2	140	17 [12-24]	-	0.0	0.0	1.00	1	0.15^{NS}
6b.	Karnataka	2011-2015	6	1,165	18 [11-28]	3-63	92.6	0.452	3.68	5	70.9**
6c.	Kerala	2016	1	31	26	-	-	-	-	-	-
6d.	Tamil Nadu	2010-2015	6	471	12 [7-22]	1-58	80.9	0.562	2.29	5	35.0**
7.	Central zone	2005 & 2007	2	196	22 [17-29]	-	23.1	0.018	1.14	1	2.61^{NS}
7a.	Chhattisgarh	2007	1	110	18	-	-	-	-	-	-
7b.	Madhya Pradesh	2005	1	86	28	-	-	-	-	-	-
8a.	Cattle	2005-2018	45	8,917	17 [14-21]	4-48	93.3	0.528	3.85	44	615.9**
8b.	Buffaloes	1995-2018	12	3,397	24 [19-30]	11-45	77.9	0.152	2.13	11	83.8**
9a.	Cultural & biochemical tests	1995-2018	51	11,355	18 [16-22]	6-46	93.6	0.426	3.95	50	678.6**
9b.	Molecular methods	2010-2018	6	959	18 [9-31]	1-77	94.5	0.826	4.28	5	110.4**
10a.	*St. agalactiae*	1995-2018	15	1,681	13 [9-18]	3-44	86.2	0.557	2.69	14	140.4**
10b.	*St. dysgalactiae*	1995-2017	8	858	15 [9-25]	2-55	88.0	0.534	2.88	7	44.3**
10c.	*St. pyogenes*	1995	1	40	5	-	-	-	-	-	-
10d.	*St. saprophyticus*	2018	1	51	4	-	-	-	-	-	-
10e.	*St. uberis*	1995-2017	8	747	2 [2-4]	1-4	0.0	0.0	1.00	7	0.79^{NS}
10f.	*St. species*	2005-2018	43	10,648	18 [15-22]	5-50	95.2	0.540	4.56	42	729.2**
11a.	Subclinical mastitis	2006-2017	30	3,483	19 [16-23]	6-44	85.5	0.343	2.63	29	190.7**
11b.	Clinical mastitis	1995-2018	8	904	19 [12-28]	4-57	87.9	0.441	2.87	7	44.7**
11c.	Mastitis	2007-2018	19	7,927	18 [13-24]	3-56	97.4	0.671	6.22	18	452.6**

Note: CI-Confidence Interval, PI-Prediction Interval, NS-Not significant, *-Significant, **- Highly Significant ($P<0.01$).

Table S5. *Escherichia coli* prevalence estimates in India based on various subgroup meta-analyses

No.	Categories	Period	Number of studies	Total samples	Pooled estimates		Tests for Heterogeneity				
					Prevalence (%) [CI at 95% level]	PI (%) at 95% level	I^2 Value (%)	Tau square value	H value	Degrees of Freedom	Cochran Q value
1.	India	1995-2018	54	12,288	15 [13-18]	4-44	92.6	0.522	3.67	53	510.5**
2a.	India-Period I	1995-2010	13	4,600	13 [10-18]	4-35	86.2	0.298	2.69	12	64.4**
2b.	India-Period II	2011-2018	41	7,688	16 [13-20]	4-48	92.1	0.565	3.55	40	440.8**
3.	North zone	1995-2018	21	8,176	14 [10-19]	3-46	94.8	0.583	4.39	20	223.2**
3a.	Haryana	2007-2018	7	6,797	15 [11-21]	4-41	94.4	0.238	4.21	6	84.5**
3b.	Jammu and Kashmir	2005-2017	6	662	13 [5-30]	0-86	92.9	1.542	3.74	5	83.5**
3c.	Punjab	1995-2018	3	266	21 [17-27]	4-65	0.0	0.0	1.00	2	4.65^{NS}
3d.	Uttarakhand	2018	1	142	3	-	-	-	-	-	-
3e.	Uttar Pradesh	2010-2018	4	309	17 [13-21]	10-28	0.0	0.0	1.00	3	1.88^{NS}

4.	East zone	2010-2017	6	878	16 [12-21]	6-36	68.5	0.124	1.78	5	18.7**
4a.	Assam	2017	1	18	39	-	-	-	-	-	-
4b.	Jharkhand	2011-2015	3	440	11 [9-15]	2-46	0.0	0.0	1.00	2	2.4NS
4c.	Odisha	2013	1	144	21	-	-	-	-	-	-
4d.	Sikkim	2010	1	276	18	-	-	-	-	-	-
5.	West zone	2009-2016	8	735	15 [9-25]	2-59	87.4	0.637	2.82	7	58.6**
5a.	Gujarat	2012 & 2016	2	259	11 [8-15]	-	0.0	0.0	1.00	1	0.84NS
5b.	Maharashtra	2009 & 2010	2	141	14 [4-41]	-	74.4	0.836	1.98	1	11.4**
5c.	Rajasthan	2010-2015	4	335	17 [7-34]	0-95	89.5	0.839	3.08	3	35.5**
6.	South zone	2009-2017	17	2,303	16 [12-23]	3-52	91.8	0.611	3.49	16	141.5**
6a.	Andhra Pradesh	2009 & 2015	2	140	11 [4-28]	-	69.8	0.499	1.82	1	8.05**
6b.	Karnataka	2011-2015	7	1,557	19 [15-25]	8-41	83.2	0.149	2.44	6	37.7**
6c.	Kerala	2016	1	31	42	-	-	-	-	-	-
6d.	Tamil Nadu	2010-2017	7	575	13 [6-25]	1-70	89.3	1.001	3.06	6	76.5**
7.	Central zone	2005 & 2007	2	196	16 [11-22]	-	0.0	0.0	1.00	1	0.30NS
7a.	Chhattisgarh	2007	1	110	15	-	-	-	-	-	-
7b.	Madhya Pradesh	2005	1	86	17	-	-	-	-	-	-
8a.	Cattle	2005-2018	46	9,059	16 [13-20]	4-48	92.8	0.581	3.72	45	407.3**
8b.	Buffaloes	1995-2018	8	3,229	11 [9-15]	5-22	60.1	0.087	1.58	7	21.5**
9a.	Cultural and biochemical tests	1995-2018	47	10,937	15 [12-18]	4-45	92.9	0.583	3.75	46	426.0**
9b.	Molecular methods	2010-2018	7	1,351	20 [16-26]	8-42	80.3	0.141	2.25	6	32.8**
10a.	Subclinical mastitis	2005-2017	22	2,689	12 [9-16]	3-36	85.8	0.464	2.66	21	122.7**
10b.	Clinical mastitis	1995-2018	11	1,208	16 [11-23]	4-50	86.5	0.465	2.72	10	65.5**
10c.	Mastitis	2007-2018	21	8,391	20 [15-25]	5-51	94.3	0.447	4.18	20	306.1**

Note: CI-Confidence Interval, PI-Prediction Interval, NS-Not significant, *-Significant (P<0.05), **- Highly Significant (P<0.01).
