# Supplementary material for: An Understanding of the Global Status of Major Bacterial Pathogens of Milk Concerning Bovine Mastitis: A Systematic Review and Meta-Analysis (Scientometrics)
Source: Pathogens. 2021 Apr 30;10(5):545. doi: 10.3390/pathogens10050545 (PMC8147236; doi:10.3390/pathogens10050545)
Supplement: Supplementary file 1 [file pathogens-10-00545-s001.zip › Supplementary files/Supplementary file S3 Reference list.pdf]

References

1. Abdel-Rady A, Sayed M. Epidemiological studies on subclinical mastitis in dairy cows in Assiut Governorate. Vet World. 2009; 2(10):373-80.
2. Abd-Elrahman AH. Mastitis in housed dairy buffaloes: incidence, etiology, clinical finding, antimicrobial sensitivity and different medical treatment against *E. coli* mastitis. Life Sci J. 2013; 10:532-8.
3. Abdulkadhim MH. Prevalence of methicillin resistance *Staphylococcus aureus* in cattle and she-camels milk at Al-Qadisyia Province, Al-Anbar. J Vet Sci. 2012; 5(2):63-7.
4. Abebe R, Hatiya H, Abera M, Megersa B, Asmare K. Bovine mastitis: prevalence, risk factors and isolation of *Staphylococcus aureus* in dairy herds at Hawassa milk shed, south Ethiopia. BMC Vet Res. 2016; 12(1):270.
5. Abera B, Lemma D, Iticha I. Study of bovine mastitis in Asella government dairy farm of Oromia Regional state, south eastern Ethiopia. Int J Curr Res Aca Rev. 2013; 1(2):134-45.
6. Abera M, Elias B, Aragaw K, Denberga Y, Amenu K, Sheferaw D. Major causes of mastitis and associated risk factors in smallholder dairy cows in Shashemene, southern Ethiopia. Afr J Agric Res. 2012; 7(24):3513-8.
7. Abo-Shama UH. Prevalence and antimicrobial susceptibility of *Staphylococcus aureus* isolated from cattle, buffalo, sheep and goats raw milk in Sohag Governorate, Egypt. Assiut Vet Med J. 2014; 60(141):63-72.
8. Abunna F, Fufa G, Megersa B, Regassa A. Bovine mastitis: prevalence, risk factors and bacterial isolation in small-holder dairy farms in Addis Ababa City, Ethiopia. Glob Vet. 2013; 10(6):647-52.
9. Adane B, Guyo K, Tekle Y, Taddele H, Bogale A, Biffa D. Study on prevalence and risk factors of bovine mastitis in Borana pastoral and agro-pastoral settings of Yabello district, Borana zone, southern Ethiopia. Am Eurasian J Agric Environ Sci. 2012; 12(10):1274-81.
10. Adesiyun AA, Webb LA, Romain HT. Prevalence and characteristics of *Staphylococcus aureus* strains isolated from bulk and composite milk and cattle handlers. J Food Prot. 1998; 61(5):629-32.
11. Ahmed HF, Straubinger RK, Hegazy YM, Ibrahim S. Subclinical mastitis in dairy cattle and buffaloes among small holders in Egypt: Prevalence and evidence of virulence of *Escherichia coli* causative agent. Trop Biomed. 2018; 35(2):321-9.
12. Akkou M, Bouchiat C, Antri K, Bes M, Tristan A, Dauwalder O, Martins-Simoes P, Rasigade JP, Etienne J, Vandenesch F, Ramdani-Bouguessa N. New host shift from human to cows within *Staphylococcus aureus* involved in bovine mastitis and nasal carriage of animal's caretakers. Vet Microbiol. 2018; 223:173-80.
13. Alekish MO. The association between the somatic cell count and isolated microorganisms during subclinical mastitis in heifers in Jordan. Vet Med. 2015; 60(2):71-6.
14. Ali MA, Ahmad MD, Muhammad K, Anjum AA. Prevalence of sub clinical mastitis in dairy buffaloes of Punjab, Pakistan. J Anim Plant Sci. 2011; 21(3):477-80.
15. Ali Z, Dimri U, Jhambh R. Prevalence and antibiogram of bacterial pathogens from subclinical mastitis in buffaloes. Buffalo Bull. 2015; 34(1):41-4.
16. Amer S, Gálvez FLA, Fukuda Y, Chika TADA, Jimenez IL, Valle WFM, Nakai Y. Prevalence and etiology of mastitis in dairy cattle in El Oro Province, Ecuador. J Vet Med Sci. 2018; 80(6):861-8.
17. Amin AS, Hamouda RH, Abdel-All AA. PCR assays for detecting major pathogens of mastitis in milk samples. World J Dairy Food Sci. 2011; 6(2): 199-206.
18. Amin B, Deneke Y, Abdela N. Bovine mastitis: prevalence, risk factors and isolation of *Streptoccocus* species from small holders dairy farms in and around Haramaya town, eastern Ethiopia. Glob J Med Res. 2017; 17(1):27-38.
19. Amosun EA, Ajuwape ATP, Adetosoye AI. Bovine streptococcal mastitis in southwest and northern states of Nigeria. Afr J Biomed Res. 2010; 13(1):33-7.

20. Anderson KL, Lyman R, Moury K, Ray D, Watson DW, Correa MT. Molecular epidemiology of *Staphylococcus aureus* mastitis in dairy heifers. J Dairy Sci. 2011; 95(9):4921-30.
21. Atyabi N, Vodjgani M, Gharahgouzlou F, Bahonar A. Prevalence of bacterial mastitis in cattle from the farms around Tehran. Iran J Vet Res. 2006; 7(3):76-9.
22. Ayano AA, Hiriko F, Simyalew AM, Yohannes A. Prevalence of subclinical mastitis in lactating cows in selected commercial dairy farms of Holeta district. J Vet Med Anim Health. 2013; 5(3):67-72.
23. Ayyappadas MP, Renugadevi R. Study on the prevalence, incidence, drug sensitivity and extra chromosomal impact on bovine mastitis pathogens. J Agric Vet Sci. 2013; 2(5):60-2.
24. Bakir M, Sabrina R, Toufik M. Antibacterial susceptibility profiles of sub-clinical mastitis pathogens isolated from cows in Batna and Setif governorates (East of Algeria). Vet World. 2011; 4(12):537-41.
25. Bakken G. Subclinical mastitis in Norwegian dairy cows: prevalence rates and epidemiological assessments. Acta agr scand. 1981; 31(3):273-86.
26. Bal EBB, Bayar S, Bal MA. Antimicrobial susceptibilities of coagulase-negative *Staphylococci* (CNS) and *Streptococci* from bovine subclinical mastitis cases. J Microbiol. 2010; 48(3):267-74.
27. Baloch H, Rind R, Shah G, Kalhoro DH, Kalhora AB. Study of clinical mastitis in buffaloes caused by Staphylococcal species. Pak J Agric Eng Vet Sci. 2013; 29(1):88-95.
28. Bansal BK, Gupta DK, Shafi TA, Sharma S. Comparative antibiogram of coagulase-negative *Staphylococci* (CNS) associated with subclinical and clinical mastitis in dairy cows. Vet World. 2015; 8(3):421-6.
29. Belayneh R, Belihu K, Wubete A. Dairy cows mastitis survey in Adama town, Ethiopia. J Vet Med Anim Health. 2013; 5(10):281-7.
30. Bengtsson B, Unnerstad HE, Ekman T, Artursson K, Nilsson-Öst M, Waller KP. Antimicrobial susceptibility of udder pathogens from cases of acute clinical mastitis in dairy cows. Vet Microbiol. 2009; 136(1-2):142-9.
31. Benhamed N, Moulay M, Aggad H, Henni J.E, Kihal M. Prevalence of mastitis infection and identification of causing bacteria in cattle in the Oran region west Algeria. J Anim Vet Adv. 2011; 10(22):3002-5.
32. Bhanot V, Chaudhri SS, Bisla RS, Singh H. Retrospective study on prevalence and antibiogram of mastitis in cows and buffaloes of eastern Haryana. Indian J Anim Res. 2012; 46(2):160-3.
33. Bharathy S, Gunaseelan L, Porteen K, Bojiraj M. Prevalence of *Staphylococcus aureus* in raw milk: Can it be a potential public health threat. Int J Adv Res. 2015; 3(2):801-6.
34. Bhat AM, Soodan JS, Singh R, Dhobi IA, Hussain T, Dar MY, Mir M. Incidence of bovine clinical mastitis in Jammu region and antibiogram of isolated pathogens. Vet Microbiol. 2017; 10(8):984-9.
35. Bhikane AU, Awandkar SP, Hase PB, Syed AM, Ghoke SS, Awaz KB. Prevalence, etiology and antibiogram of subclinical mastitis in crossbred cows. Vet Pract. 2010; 11(2):122-3.
36. Bi Y, Wang YJ, Qin Y, Guix Vallverdú R, Maldonado García J, Sun W, Li S, Cao Z. Prevalence of bovine mastitis pathogens in bulk tank milk in China. PLoS One. 2016; 11(5):1-13.
37. Birhanu M, Leta S, Mamo G, Tesfaye S. Prevalence of bovine subclinical mastitis and isolation of its major causes in Bishoftu Town, Ethiopia. BMC Res Notes. 2017; 10(1):767.
38. Bortolami A, Gianesella M, Fiore E, Corro M, Catania S, Morgante M. Evaluation of the udder health status in subclinical mastitis affected dairy cows through bacteriological

culture, somatic cell count and thermographic imaging. Pol J Vet Sci. 2015; 18(4):799-805.
39. Boynukara B, Gulhan T, Alisarli M, Gurturk K, Solmaz H. Classical enterotoxigenic characteristics of *Staphylococcus aureus* strains isolated from bovine subclinical mastitis in Van, Turkey. Int J Food Microbiol. 2008; 125(2):209-11.
40. Bradley AJ, Green MJ. Aetiology of clinical mastitis in six Somerset dairy herds. Vet Rec. 2001; 148(22):683-6.
41. Breen JE, Green MJ, Bradley AJ. Quarter and cow risk factors associated with the occurrence of clinical mastitis in dairy cows in the United Kingdom. J Dairy Sci. 2009; 92(6):2551-561.
42. Brooks BW, Barnum DA, Meek AH. A survey of mastitis in selected Ontario dairy herds. Can Vet J. 1982; 23(5):156.
43. Budri PE, Silva NC, Bonsaglia EC, Júnior AF, Júnior, JA, Doyama JT, Gonçalves JL, Santos MV, Fitzgerald-Hughes D, Rall VL. Effect of essential oils of *Syzygium aromaticum* and *Cinnamomum zeylanicum* and their major components on biofilm production in *Staphylococcus aureus* strains isolated from milk of cows with mastitis. J Dairy Sci. 2015; 98(9):5899-904.
44. Bulla TR, Rana YS, Sharma A, Beniwal BS. Prevalence of subclinical mastitis in Murrah buffaloes. Haryana Vet. 2006; 45:53-6.
45. Cao LT, Wu JQ, Xie F, Hu SH, Mo Y. Efficacy of nisin in treatment of clinical mastitis in lactating dairy cows. J Dairy Sci. 2007; 90(8):3980-5.
46. Ceniti C, Britti D, Santoro AML, Musarella R, Ciambrone L, Casalinuovo F, Costanzo N. Phenotypic antimicrobial resistance profile of isolates causing clinical mastitis in dairy animals. Ital J Food Saf. 2017; 6(2):84-7.
47. Cervinkova D, Vlkova H, Borodacova I, Makovcova J, Babak V, Lorencova A, Vrtkova I, Marosevic D, Jaglic Z. Prevalence of mastitis pathogens in milk from clinically healthy cows. Vet Med. 2013; 58(11):567-75.
48. Charaya G, Sharma A, Kumar A, Singh M, Goel P. Pathogens isolated from clinical mastitis in Murrah buffaloes and their antibiogram. Vet World. 2014; 7(11):980-85.
49. Charaya G, Sharma A, Singh M, Tiwari S, Pankaj KA. 2013. Subclinical mastitis at an organised farm: prevalence, etiology and antibiogram. Haryana Vet. 52:30-2.
50. Cheng J, Qu W, Barkema HW, Nobrega DB, Gao J, Liu G, De Buck J, Kastelic JP, Sun H, Han B. Antimicrobial resistance profiles of 5 common bovine mastitis pathogens in large Chinese dairy herds. J Dairy Sci. 2019; 102(3):2416-26.
51. Condas LA, De Buck J, Nobrega DB, Carson DA, Naushad S, De Vliegher S, Zadoks RN, Middleton JR, Dufour S, Kastelic JP, Barkema HW. Prevalence of non-aureus Staphylococci species causing intramammary infections in Canadian dairy herds. J Dairy Sci. 2017; 100(7):5592-612.
52. Daniel RCW, O'boyle D, Marek MS, Frost AJ. A survey of clinical mastitis in south-east Queensland dairy herds. Aust Vet J. 1982; 58(4):143-7.
53. Dar KH, Ansari MM, Dar SH, Tantary HA, Baba MA, Mehraj-ud-Din N. Studies on subclinical mastitis in dairy cows of Jammu and Kashmir. Int J Vet Sci. 2014; 3(2):95-9.
54. Das G, Lalnunpuia C, Sarma K, Behera SK, Dutta TK, Bandyopadhyay S. Prevalence of *Staphylococcus aureus* associated sub-clinical mastitis in crossbred cows in Mizoram. Ruminant Sci. 2015; 4(2):167-70.
55. Das PK, Joseph E. Identification and antibigram of microbes associated with buffalo mastitis in Jabalpur, Madhya Pradesh, India. Buffalo Bull. 2005; 24:3-9.
56. Davies PL, Leigh JA, Bradley AJ, Archer SC, Emes RD, Green MJ. Molecular epidemiology of *Streptococcus uberis* clinical mastitis in dairy herds: strain heterogeneity and transmission. J Clin Microbiol. 2015; 54(1):68-74.

57. Dego OK, Tareke F. Bovine mastitis in selected areas of southern Ethiopia. Trop Anim Health Prod. 2003; 35(3):197-205.
58. Demme B, Abegaz S. Isolation and identification of major bacterial pathogen from clinical mastitis cow raw milk in Addis Ababa, Ethiopia. Acad J Anim Dis. 2015; 4:44-51.
59. Dieser SA, Vissio C, Lasagno MC, Bogni CI, Larriestra AJ, Odierno LM. Prevalence of pathogens causing subclinical mastitis in Argentinean dairy herds. Pak Vet J. 2014; 34(1):124-6.
60. Döpfer D, Barkema HW, Lam TJGM, Schukken YH, Gaastra W. Recurrent clinical mastitis caused by *Escherichia coli* in dairy cows. J Dairy Sci. 1998; 82(1):80-5.
61. Dubal ZB, Rahman H, Papri P, Kumar A, Kalpana P. Characterization and antimicrobial sensitivity of the pathogens isolated from bovine mastitis with special reference to *Escherichia coli* and Staphylococcus species. Indian J Anim Sci. 2010; 80(12):1163-7.
62. Duguma A, Tolosa T, Yohannes A. Prevalence of clinical and sub-clinical mastitis on cross bred dairy cows at Holleta Agricultural Research Center, Central Ethiopia. J Vet Med Anim Health. 2014; 6(1):13-7.
63. Edinger D, Tenhagen BA, Kalbe P, Klünder G, Baumgärtner B, Heuwieser W. Effect of teat dipping with a germicide barrier teat dip in late gestation on intramammary infection and clinical mastitis during the first 5 days post-partum in primiparous cows. J Vet Med-A. 2000; 47(8):463-8.
64. Elango A, Doraisamy KA, Rajarajan G, Kumaresan G. Bacteriology of sub clinical mastitis and antibiogram of isolates recovered from cross bred cows. Indian J Anim Res. 2010; 44(4):280-4.
65. Elbably MA, Emeash HH, Asmaa NM. Risk factors associated with mastitis occurrence in dairy herds in Benisuef, Egypt. World's Vet J. 2013; 3(1):5-10.
66. Elemo KK, Sisay T, Shiferaw A, Fato MA. Prevalence, risk factors and multidrug resistance profile of *Staphylococcus aureus* isolated from bovine mastitis in selected dairy farms in and around Asella town, Arsi Zone, south eastern Ethiopia. Afr J Microbiol Res. 2017; 11(45):1632-42.
67. Elhaig MM, Selim A. Molecular and bacteriological investigation of subclinical mastitis caused by *Staphylococcus aureus* and *Streptococcus agalactiae* in domestic bovids from Ismailia, Egypt. Trop Anim Health Prod. 2014; 47(2):271-6.
68. El-Jakee JK, Aref NE, Gomaa A, El-Hariri MD, Galal HM, Omar SA, Samir A. Emerging of coagulase negative Staphylococci as a cause of mastitis in dairy animals: An environmental hazard. Int J Vet Sci Med. 2013; 1(2):74-8.
69. Elsayed MS, Abd Elrahman Mahmoud El-Bagoury M, Dawoud A. Phenotypic and genotypic detection of virulence factors of *Staphylococcus aureus* isolated from clinical and subclinical mastitis in cattle and water buffaloes from different farms of Sadat City in Egypt. Vet World. 2015; 8(9):1051-8.
70. Erskine RJ, Bartlett PC, VanLente JL, Phipps CR. Efficacy of systemic ceftiofur as a therapy for severe clinical mastitis in dairy cattle. J Dairy Sci. 2002; 85(10):2571-5.
71. Farooq AA, Inayat S, Akhtar MS, Mushtaq M. Prevalence of mastitis and antibiotic sensitivity of bacterial isolates recovered from Nili-Ravi buffaloes. J Anim Plant Sci. 2008; 18(2-3):76-7.
72. Freitas DMF, Luz IDS, Silveira-Filho VDM, WP Júnior J, Stamford TL, Mota RA, Sena MJD, de Almeida AM, Balbino VDQ, Leal-Balbino TC. Staphylococcal toxin genes in strains isolated from cows with subclinical mastitis. Pesqui Vet Bras. 2008; 28(12):617-21.
73. Ganai AW, Kotwal SK, Malik MA, Sharma HK, Wani N, Jeelani R. Prevalence of *Staphylococcus aureus* and methicillin resistant *Staphylococcus aureus* (MRSA) in clinical setting and dairy farm environment of Jammu. J Anim Res. 2015; 5(3):539-45.

74. Ganda EK, Bisinotto RS, Decter DH, Bicalho RC. Evaluation of an on-farm culture system (Accumast) for fast identification of milk pathogens associated with clinical mastitis in dairy cows. PloS One. 2016; 11(5):1-16.
75. Gao J, Barkema HW, Zhang L, Liu G, Deng Z, Cai L, Shan R, Zhang S, Zou J, Kastelic JP, Han B. Incidence of clinical mastitis and distribution of pathogens on large Chinese dairy farms. J Dairy Sci. 2017; 100(6):4797-806.
76. Getahun K, Kelay B, Bekana M, Lobago F. Bovine mastitis and antibiotic resistance patterns in Selalle smallholder dairy farms, central Ethiopia. Trop Anim Health Prod. 2008; 40(4):261-8.
77. Gillespie BE, Headrick SI, Boonyayatra S, Oliver SP. Prevalence and persistence of coagulase-negative Staphylococcus species in three dairy research herds. Vet Microbiol. 2009; 134(1-2):65-72.
78. Gogoi SM, Tamuly U, Khuman LS. Prevalence of sub-clinical mastitis in areas around Lakhimpur town of Assam. Int J Agric Sci Res. 2017; 7(1):501-8.
79. Gonzalez RN, Giraudo JA, Busso JJ. Studies on subclinical mastitis. II. Bacterial agents. Rev Med Vet Arg. 1980; 61(3):225-34.
80. Graber HU, Naskova J, Studer E, Kaufmann T, Kirchhofer M, Brechbühl M, Schaeren W, Steiner A, Fournier C. Mastitis-related subtypes of bovine *Staphylococcus aureus* are characterized by different clinical properties. J Dairy Sci. 2009; 92(4):1442-51.
81. Green MJ, Green LE, Medley GF, Schukken YH, Bradley AJ. Influence of dry period bacterial intramammary infection on clinical mastitis in dairy cows. J Dairy Sci. 2002; 85(10):2589-99.
82. Guha A, Gera S. Etio-prevalence of sub clinical mastitis in Holstein x Haryana crossbred cattle. Explor Anim Med Res. 2011; 1(1):75-8.
83. Guha A, Guha R, Gera S. Comparison of somatic cell count, California mastitis test, chloride test and rennet coagulation time with bacterial culture examination to detect subclinical mastitis in riverine buffalo (*Bubalus bubalis*). Afr J Agric Res. 2012; 7(41):5578-84.
84. Gupta N, Faridi F, Maherchandani S, Kashyap SK. Detection and genetic characterization of major pathogens isolated from bovine mastitis in Rajasthan. Ind J Vet Sci Biotech. 2015; 11(2):1-6.
85. Haftu R, Taddele H, Gugsa G, Kalayou S. Prevalence, bacterial causes, and antimicrobial susceptibility profile of mastitis isolates from cows in large-scale dairy farms of northern Ethiopia. Trop Anim Health Prod. 2012; 44(7):1765-71.
86. Haghkhah M, Ahmadi MR, Gheisari HR, Kadivar A. Preliminary bacterial study on subclinical mastitis and teat condition in dairy herds around Shiraz. Turk J Vet Anim Sci. 2011; 35(6):387-94.
87. Haghkhah M, Nazifi S, Jahromi AG. Evaluation of milk haptoglobin and amyloid A in high producing dairy cattle with clinical and subclinical mastitis in Shiraz. Comp Clin Path. 2009; 19(6):547-52.
88. Hagnestam C, Emanuelson U, Berglund B. Yield losses associated with clinical mastitis occurring in different weeks of lactation. 2007; J Dairy Sci. 90(5):2260-70.
89. Hailemeskel D, Admasu P, Alemu F. Prevalence and identification of bacterial pathogens causing bovine mastitis from crossbred of dairy cows in north Showa Zone of Ethiopia. Glob Vet. 2014; 13(2):189-95.
90. Hamed MI, Ziatoun AMA. Prevalence of *Staphylococcus aureus* subclinical mastitis in dairy buffaloes' farms. Int J Livest Res. 2014; 4(3):21-8.
91. Hameed KGA, Sender G, Korwin-Kossakowska A. Public health hazard due to mastitis in dairy cows. Anim Sci Pap Rep. 2006; 25(2):73-85.

92. Harini H, Sumathi BR. Screening of bovine milk samples for sub-clinical mastitis and antibiogram of bacterial isolates. Vet World. 2011; 4(8):358-9.
93. Harjanti DW, Ciptaningtyas R, Wahyono F, Setiatin DET. Isolation and identification of bacterial pathogen from mastitis milk in Central Java Indonesia. Int Sym Food Agro-biodiv. 2018; 1-6.
94. Hashemi M, Kafi M, Safdarian M. The prevalence of clinical and subclinical mastitis in dairy cows in the central region of Fars province, south of Iran. Iran J Vet Res. 2011; 12(3):236-41.
95. Hegde R, Isloor S, Prabhu KN, Shome BR, Rathnamma D, Suryanarayana VVS, Yatiraj S, Prasad CR, Krishnaveni N, Sundareshan S, Akhila DS. Incidence of subclinical mastitis and prevalence of major mastitis pathogens in organized farms and unorganized sectors. Indian J Microbiol. 2013; 53(3):315-20.
96. Hussein SA. Prevalence and bacterial etiology of subclinical mastitis in dairy cows in Al Sulaimaniyah district. Kufa J Vet Sci. 2012; 3(1):190-203.
97. Idriss SE, Foltys V, Tančin V, Kirchnerová K, Zaujec K. Mastitis pathogens in milk of dairy cows in Slovakia. Slovak J Anim Sci. 2013; 46(3):115-9.
98. Iraguha B, Hamudikuwanda H, Mushonga B. Bovine mastitis prevalence and associated risk factors in dairy cows in Nyagatare District, Rwanda. J S Afr Vet Assoc. 2015; 86(1):1-6.
99. Islam NN, Farzana Z, Chowdhury AMMA, Mannan A, Kamaruddin KM, Siddiki AMAMZ, Uddin I. Characterization of bovine subclinical mastitis caused by *Staphylococcus aureus* in southern Bangladesh by bacteriological and molecular approaches. Asian J Biol Sci. 2014; 7:1-12.
100. Jamali H, Radmehr B, Ismail S. Prevalence and antibiotic resistance of *Staphylococcus aureus* isolated from bovine clinical mastitis. J Dairy Sci. 2014; 97(4):2226-30.
101. Jena B, Pagrut NK, Sahoo A, Ahmed A. Subclinical bovine mastitis in rural, peri-urban and suburban regions of Jaipur district of Rajasthan, India. J Anim Res. 2015; 5(1):175-82.
102. Jeykumar M, Vinodkumar G, Bashir BP, Krovvidi S. Antibiogram of mastitis pathogens in the milk of crossbred cows in Namakkal district, Tamil Nadu. Vet World. 2013; 6(6):354-6.
103. Joshi H, Tanwar RK, Chahar A. Prevalence of sub-clinical mastitis in buffaloes. Vet Pract. 2013; 14(2):377-8.
104. Junaidu AU, Salihu MD, Tambuwal FM, Magaji AA, Jaafaru S. Prevalence of mastitis in lactating cows in some selected commercial dairy farms in Sokoto metropolis. Adv Appl Sci Res. 2011; 2(2):290-4.
105. Kalantari A, Safi S, Foroushani AR. Milk lactate dehydrogenase and alkaline phosphatase as biomarkers in detection of bovine subclinical mastitis. Ann Biol Res. 2013; 4(2):302-7.
106. Kaliwal BB, Sadashiv SO, Kurjogi MM, Sanakal RD. Prevalence and antimicrobial susceptibility of coagulase-negative Staphylococci isolated from bovine mastitis. Vet World. 2011; 4(4):158-61.
107. Kalmus P, Viltrop A, Aasmäe B, Kask K. Occurrence of clinical mastitis in primiparous Estonian dairy cows in different housing conditions. Acta Vet Scand. 2006; 48(1):21.
108. Karabasanavar NS, Radder SK, Sivaraman GK. Field level interventions on subclinical mastitis and detection of *Staphylococcus* in crossbred dairy cows. Indian J Anim Sci. 2019; 89(7):711-7.
109. Kavitha KL, Rajesh K, Suresh K, Satheesh K, Sundar NS. Buffalo mastitis-risk factors. Buffalo Bull. 2009; 28(3):134-7.

110. Kayesh MEH, Talukder M, Anower AKMM. Prevalence of subclinical mastitis and its association with bacteria and risk factors in lactating cows of Barisal district in Bangladesh. Int J Biol Res. 2014; 2(2):35-8.
111. Kedir J, Disassa H, Jaleta H, Zenebe T, Kebede G. A Study on bovine mastitis, isolation and identification of Staphylococcus species in dairy farms of Dire Dawa city, eastern Ethiopia. Glob Vet. 2016; 16(3):222-30.
112. Kirkan Ş, Göksoy EÖ, Kaya O. Identification and antimicrobial susceptibility of *Staphylococcus aureus* and coagulase negative Staphylococci from bovine mastitis in the Aydın region of Turkey. Turkish J Vet Anim Sci. 2003; 29(3):791-6.
113. Kivaria FM, Noordhuizen JPTM, Kapaga AM. Evaluation of the hygienic quality and associated public health hazards of raw milk marketed by smallholder dairy producers in the Dar Salaam region, Tanzania. Trop Anim Health Pro. 2006; 38(3):185-94.
114. Klimienė I, Ružauskas M, Špakauskas V, Mockeliūnas R, Pereckienė A, Butrimaitė-Ambrozevičienė Č. Prevalence of gram positive bacteria in cow mastitis and their susceptibility to beta-lactam antibiotics. Vet ir Zootech. 2011; 56(78):65-72.
115. Kour G, Chandra M, Kaur G, Narang D, Gupta DK, Arora AK, Sharma NS. Prevalence of mastitis causing organism and their antibiotic resistance pattern in dairy farms. Indian J Dairy Sci. 2017; 70(5):587-92.
116. Krishnaveni N, Isloor SK, Hegde R, Suryanarayanan VVS, Rathnma D, Veeregowda BM, Nagaraja CS, Sundareshan S. Rapid detection of virulence associated genes in Streptococcal isolates from bovine mastitis. Afr J Microbiol Res. 2014; 8(22):2245-54.
117. Krukowski H, Tietze M, Majewski T, Różański P. Survey of yeast mastitis in dairy herds of small-type farms in the Lublin region, Poland. Mycopathologia. 2000; 150(1):5-7.
118. Kudinha T, Simango C. Prevalence of coagulase-negative staphylococci in bovine mastitis in Zimbabwe. J S Afr Vet Assoc. 2002; 73(2):62-5.
119. Kumar A, Anu R, Dwivedi SK, Gupta MK. Bacterial prevalence and antibiotic resistance profile from bovine mastitis in Mathura, India. Egypt J Dairy Sci. 2010; 38(1):31-4.
120. Kumar A, Haque S, Singh KK, Roy BK. Bacteriology and antibiogram of bovine mastitis in Ranchi and its vicinity. Int J Environ Sci Technol. 2015; 4:1066-72.
121. Kurjogi MM, Kaliwal BB. Prevalence and antimicrobial susceptibility of bacteria isolated from bovine mastitis. Adv Appl Sci Res. 2011; 2(6):229-35.
122. Lafi S, Al-Rawashdeh O, Na'Was T, Hailat N. National cross-sectional study of mastitis in dairy cattle in Jordan. Trop Anim Health Pro. 1994; 26(3):168-74.
123. Lago A, Godden SM, Bey R, Ruegg PL, Leslie K. The selective treatment of clinical mastitis based on on-farm culture results: I. Effects on antibiotic use, milk withholding time, and short-term clinical and bacteriological outcomes. J Dairy Sci. 2011; 94(9):4441-56.
124. Lakew M, Tolosa T, Tigre W. Prevalence and major bacterial causes of bovine mastitis in Asella, south eastern Ethiopia. Trop Anim Health Pro. 2009; 41(7):1525-30.
125. Lakshmi R, Jayavardhanan K. Isolation and identification of major causing bacteria from bovine mastitis. Int J Appl Pure Sci Agric. 2016; 2(4):45-8.
126. Lamey AE, Ammar AM, Zaki ER, Khairy N, Moshref BS, Refai MK. Virulence factors of *Escherichia coli* isolated from recurrent cases of clinical and subclinical mastitis in buffaloes. Int J Microbiol Res. 2013; 4(1):86-94.
127. Lasagno MC, Reinoso EB, Dieser SA, Calvinho LF, Buzzola F, Vissio C, Bogni CI, Odierno LM. Phenotypic and genotypic characterization of *Streptococcus uberis* isolated from bovine subclinical mastitis in Argentinean dairy farms. Rev Argent Microbiol. 2011; 43(3):212-7.
128. León-Galván M, Barboza-Corona JE, Lechuga-Arana AA, Valencia-Posadas M, Aguayo DD, Cedillo-Pelaez C, Martínez-Ortega EA, Gutierrez-Chavez AJ. Molecular detection

and sensitivity to antibiotics and bacteriocins of pathogens isolated from bovine mastitis in family dairy herds of central Mexico. Biomed Res Int. 2015; 9.
129. Li JP, Zhou HJ, Yuan L, He T, Hu SH. Prevalence, genetic diversity and antimicrobial susceptibility profiles of *Staphylococcus aureus* isolated from bovine mastitis in Zhejiang Province, China. J Zhejiang Univ Sci B. 2009; 10(10):753-60.
130. Lucia M, Rahayu S, Haerah D, Wahyuni D. Detection of *Staphylococcus aureus* and *Streptococcus agalactiae*: subclinical mastitis causes in dairy cow and dairy buffalo (*Bubalus bubalis*). Am J Biomed Res. 2017; 5(1):8-13.
131. Maćešić N, Bačić G, Božičević K, Benić M, Karadjole T, Prvanović Babić N, Lojkić M, Efendić M, Bačić I, Pavlak M. Assessment of the Zagreb mastitis test in diagnosis of subclinical mastitis in dairy cattle. Vet Arh. 2016; 86(4):475-85.
132. Maćešić N, Karadjole T, Bačić G, Benić M, Karadjole M, Vince S, Lipar M, Cergolj M. Aetiology and prevalence of bovine intra mammary infection at drying off. Vet Arh. 2012; 82(2):125-31.
133. Marashifard M, Aliabad ZK, Hosseini SAAM, Darban-Sarokhalil D, Mirzaii M, Khoramrooz SS. Determination of antibiotic resistance pattern and virulence genes in *Escherichia coli* isolated from bovine with subclinical mastitis in southwest of Iran. Trop Anim Health Pro. 2019; 51(3):575-80.
134. Marija P, Stanko B, Branko V, Zoran R, Vera K, Miodrag R, Aleksandra N, Dušan S, Milijana B. Prevalence and molecular characterization of enterotoxin-producing strains of *Staphylococcus aureus* isolated from serbian dairy cows. Acta Vet. 2016; 66(4):466-77.
135. Marimuthu M, Abdullah FFJ, Mohammed K, Sangeetha D, Poshpum OS, Adamu L, Osman AY, Abba Y, Tijjani A. Prevalence and antimicrobial resistance assessment of subclinical mastitis in milk samples from selected dairy farms. Am J Anim Vet Sci. 2014; 9(1):65.
136. Mdegela RH, Karimuribo E, Kusiluka LJM, Kabula B, Manjurano A, Kapaga AM, Kambarage DM. Mastitis in smallholder dairy and pastoral cattle herds in the urban and peri-urban areas of the Dodoma municipality in Central Tanzania. Livest Res Rural Dev. 2005; 17(11):123.
137. Mdegela RH, Ryoba R, Karimuribo ED, Phiri EJ, Løken T, Reksen O, Mtengeti E, Urio NA. Prevalence of clinical and subclinical mastitis and quality of milk on smallholder dairy farms in Tanzania. J S Afr Vet Assoc. 2009; 80(3):163-8.
138. Megersa B, Manedo A, Regassa MAA, Abunna F. Mastitis in lactating cows at Hawassa Town: prevalence, risk factors, major bacterial causes and treatment response to routinely used antibiotics. Am Eurasian J Agric Environ Sci. 2012; 7(2):86-91.
139. Mekibib B, Furgasa M, Abunna F, Megersa B, Regassa A. Bovine mastitis: Prevalence, risk factors and major pathogens in dairy farms of Holeta Town, central Ethiopia. Vet World. 2010; 3(9):397-403.
140. Mekonnen H, Tesfaye A. Prevalence and etiology of mastitis and related management factors in market oriented smallholder dairy farms in Adama, Ethiopia. Rev Med Vet. 2010; 161(12):574-9.
141. Memon J, Kashif J, Yaqoob M, Liping W, Yang Y, Hongjie F. Molecular characterization and antimicrobial sensitivity of pathogens from sub-clinical and clinical mastitis in eastern China. Pak Vet J. 2013; 33(2):170-4.
142. Mesquita AA, Rocha CM, Bruhn FR, Custódio DA, Braz MS, Pinto SM, Silva DB, Costa GM. *Staphylococcus aureus* and *Streptococcus agalactiae*: prevalence, resistance to antimicrobials, and their relationship with the milk quality of dairy cattle herds in Minas Gerais state, Brazil. Pesqui Vet Bras. 2019; 39(5):308-6.
143. Michael LG, Benti D, Feyissa B, Abebe M. Study on prevalence of bovine mastitis in lactating cows and associated risk factors in and around Areka town, southern of Ethiopia. Afr J Microbiol Res. 2013; 7(43):5051-6.

144. Milne MH, Barret DC, Fitzpatrick JL, Biggs AM. Prevalence and aetiology of clinical mastitis on dairy farms in Devon. Vet Rec. 2002; 151(8):241.
145. Miltenburg JD, De Lange D, Crauwels APP, Bongers JH, Tielen MJM, Schukken YH, Elbers ARW. Incidence of clinical mastitis in a random sample of dairy herds in the southern Netherlands. Vet Rec. 1996; 139(9):204-7.
146. Mir AQ, Bansal BK, Gupta DK. Subclinical mastitis in machine milked dairy farms in Punjab: prevalence, distribution of bacteria and current antibiogram. Vet World. 2014; 7(5):291-4.
147. Mohanty NN, Das P, Pany SS, Sarangi LN, Ranabijuli S, Panda HK. Isolation and antibiogram of *Staphylococcus*, *Streptococcus* and *Escherichia coli* isolates from clinical and subclinical cases of bovine mastitis. Vet World. 2013; 6(10):739-43.
148. Momtaz H. Investigation of virulence factors in *Escherichia coli* isolated from clinical and subclinical bovine mastitis. Bulg J Vet Med. 2010; 13(2):122-6.
149. Morse D, DeLorenzo MA, Wilcox CJ, Collier RJ, Natzke RP, Bray DR. Climatic effects on occurrence of clinical mastitis. J Dairy Sci. 1988; 71(3):848-53.
150. Motamedi H, Seyfiabad SM, Ghorbanpour M, Jamshidian M, Gouraninezhad S. A polymerase chain reaction based study on the subclinical mastitis caused by *Streptococcus agalactiae S. dysgalactiae* and *S. uberis* in cattle in Ahvaz. Iran J Vet Res. 2007; 8(3):260-5.
151. Motto SK, Machang'u RS, Makondo ZK. Mastitis pathogens prevalent in dairy cattle at Magadu farm, Morogoro-Tanzania. Tanzania Vet J. 2017; 32(1):97-100.
152. Mpatswenumugabo JP, Bebora LC, Gitao GC, Mobegi VA, Iraguha B, Kamana O, Shumbusho B. Prevalence of subclinical mastitis and distribution of pathogens in dairy farms of Rubavu and Nyabihu districts, Rwanda. J Vet Med. 2017; 1-8.
153. Mubarack HM, Doss A, Vijayasanthi M, Venkataswamy R. Antimicrobial drug susceptibility of *Staphylococcus aureus* from subclinical bovine mastitis in Coimbatore, Tamilnadu, South India. Vet World, 2012; 5(6):352-5.
154. Mulate B, Abegaz S, Nazir S. Antibiogram of bacterial pathogens isolated from subclinical mastitis in Kombolcha, south Wollo, Ethiopia. Bull Anim Health Prod Afr. 2017; 65:37-47.
155. Mureithi DK, Khang KC, Kamau MN. Antimicrobial resistance profile in bacterial isolates from subclinical mastitic milk samples in dairy herds in Kenya. Bull Anim Health Prod Afr. 2017; 65:167-73.
156. Nam HM, Kim JM, Lim SK, Jang KC, Jung SC. Infectious aetiologies of mastitis on Korean dairy farms during 2008. Res Vet Sci. 2010; 88(3):372-4.
157. Navaneethan R, Saravanan S, Suresh P, Ponnuswamy KK, Palanivel KM. Prevalence of clinical mastitis due to E. coli in bovines. Int J Curr Microbiol Appl Sci. 2017; 6(10):405-9.
158. Ndahetuye JB, Persson Y, Nyman AK, Tukei M, Ongol MP, Båge R. Aetiology and prevalence of subclinical mastitis in dairy herds in peri-urban areas of Kigali in Rwanda. Trop Anim Health Pro. 2019; 51(7):2037-44.
159. Ondiek JO, Ogore PB, Shakala EK, Kaburu GM. Prevalence of bovine mastitis, its therapeutics and control in Tatton Agriculture Park, Egerton University, Njoro District of Kenya. Res J Agri Sci Rev. 2013; 2(1):15-20.
160. Östensson K, Lam V, Sjögren N, Wredle E. Prevalence of subclinical mastitis and isolated udder pathogens in dairy cows in southern Vietnam. Trop Anim Health Pro. 2013; 45(4):979-86.
161. Othman N, Bahaman AR. Prevalence of subclinical mastitis andantibiotic resistant bacteria in three selected cattle farms in Serdang, Selangorand Kluang, Johor. J Vet Malays. 2005; 17(1):27-31.

162. Pal M, Lemu D, Bilato T. Isolation, Identification and antibiogram of bacterial pathogens from bovine subclinical mastitis in Asella, Ethiopia. Int J Livest Res. 2017; 7:62-70.
163. Panahi M, Saei HD. Genetic diversity and methicillin resistance of *Staphylococcus aureus* originating from buffaloes with mastitis in Iran. Comp Immunol Microbiol Infect Dis. 2019; 62:19-24.
164. Pankaj AS, Chhabra R, Sindhu N. Prevalence of sub clinical mastitis in cows: Its etiology and antibiogram. Indian J Anim Res. 2012; 46(4):348-53.
165. Pankaj AS, Chhabra R, Sindhu N. Sub-clinical mastitis in Murrah buffaloes with special reference to prevalence, etiology and antibiogram. Buffalo Bull. 2013; 32(2):107-15.
166. Pankey JW, Drechsler PA, Wildman EE. Mastitis prevalence in primigravid heifers at parturition. J Dairy Sci. 1991; 74(5):1550-2.
167. Pardo BR, Mendoza-Sánchez G, Nader Filho A, Santos T, Langoni H, Tonhati H, Ferreira EBS, Ravena DL, Oliveira MEA, Sturion DJ. Microbiological evaluation of milk samples positive to California mastitis test in dairy buffalo cows (*Buballus bubalis*). Ital J Anim Sci. 2007; 6(2):884-7.
168. Patel JV, Bhingaradia BV, Patel BB, Patel SB, Patel PB, Vahora SP. Study on prevalence of mastitis and antibiotic sensitivity of bacterial isolates recovered from crossbred cows of Anand district of Gujarat. Indian J Dairy Sci. 2012; 65(6):467-71.
169. Patnaik S, Prasad A, Ganguly S. Biochemical characterization and antibiogram of Staphylococcal microorganisms associated with subclinical mastitis in lactating crossbred cows. Anim Sci. 2014; 8(4):123-9.
170. Paul I, Isore DP, Joardar SN, Mukhopadhayay SK, Ganguly S, Pal S. Bacteriological investigation and antibiogram on methicillin-resistant *Staphylococcus aureus* (MRSA) causing subclinical mastitis in dairy cattle population of West Bengal. Indian J Comp Microbiol Immunol Infect Dis. 2013; 34(2):56-9.
171. Perry BD, Carter ME, Hill FWG, Milne JAC. Mastitis and milk production in cattle in a communal land of Zimbabwe. Br Vet J. 1987; 143(1):44-50.
172. Petrovski KR, Heuer C, Parkinson TJ, Williamson NB. The incidence and aetiology of clinical bovine mastitis on 14 farms in Northland, New Zealand. N Z Vet J. 2009; 57(2):109-15.
173. Phuektes P, Mansell PD, Dyson RS, Hooper ND, Dick JS, Browning GF. Molecular epidemiology of *Streptococcus uberis* isolates from dairy cows with mastitis. J Clin Microbiol. 2001; 39(4):1460-6.
174. Plozza K, Lievaart JJ, Potts G, Barkema HW. Subclinical mastitis and associated risk factors on dairy farms in new south Wales. Aust Vet J. 2011; 89(1-2):41-6.
175. Prabhakar SK, Singh KB, Joshi DV. Incidence and etiology of clinical mastitis in buffaloes. Buffalo Bull. 1995; 14(3):63-7.
176. Prabhu KN, Ruban WS, Kumar GSN, Sharada R, Padalkar RD. Sub-clinical mastitis in buffaloes: prevalance, isolation and antimicrobial resistance of *Staphylococcus aureus*. Buffalo Bull. 2015; 34(2):215-22.
177. Preethirani PL, Isloor S, Sundareshan S, Nuthanalakshmi V, Deepthikiran K, Sinha AY, Rathnamma D, Nithin Prabhu K, Sharada R, Mukkur TK, Hegde NR. Isolation, biochemical and molecular identification, and in-vitro antimicrobial resistance patterns of bacteria isolated from bubaline subclinical mastitis in South India. Plos One. 2015; 10(11):1-15.
178. Pyörälä S, Hovinen M, Simojoki H, Fitzpatrick J, Eckersall PD, Orro T. Acute phase proteins in milk in naturally acquired bovine mastitis caused by different pathogens. Vet Rec. 2011; 168(535):1-7.
179. Qadri SIA, Shaheen M, Baig US, Malik H, Bhat ZI, Bhat IA, Dar PA, Yousuf RW, Iqbal RM, Bhat AM, Haq AU. Aetio-prevalence study on bovine sub clinical mastitis in lactating jersey cross-bred cows. Int J Curr Microbiol Appl Sci. 2017; 6(10):3354-7.

180. Rafiullah R, Khan MA, Shafee M, Akbar A, Ali A, Shoaib M, Ashraf F, Khan N. Occurrence of mastitis and associated pathogens with antibiogram in animal population of Peshawar, Pakistan. Thai J Vet Med. 2017; 47(1):103-8.
181. Ranjan R, Gupta MK, Singh KK. Study of bovine mastitis in different climatic conditions in Jharkhand, India. Vet World. 2011; 4(5):205-8.
182. Reza VH, Mogaddam FM, Sadegh MM, Mirzaii, H. Bacterial pathogens of intramammary infections in Azeri buffaloes of Iran and their antibiogram. Afr J Agric Res. 2011; 6(11):2516-21.
183. Sadashiv SO, Kaliwal BB. Screening and antibiotic resistance of *Escherichia coli* isolated from bovine mastitis in the region of north Karnataka, India. Indo Am J pharm Res. 2012; 5:1309-16.
184. Saidi R, Khelef D, Kaidi R. Bovine mastitis: Prevalence of bacterial pathogens and evaluation of early screening test. Afr J Microbiol Res. 2013; 7(9):777-82.
185. Saini V, McClure JT, Scholl DT, DeVries TJ, Barkema HW. Herd-level relationship between antimicrobial use and presence or absence of antimicrobial resistance in gram-negative bovine mastitis pathogens on Canadian dairy farms. J Dairy Sci. 2013; 96(8):4965-76.
186. Sanotharan N, Pagthinathan M, Nafees MSM. Prevalence of bovine subclinical mastitis and its association with bacteria and risk factors in milking cows of Batticaloa District in Sri Lanka. Int J Sci Res Innov Std. 2016; 3(6):137-50.
187. Saravanajayam M, Ravikumar R, Balasubramaniam A, Palanivel KM. Isolation of bacteria, their sensitivity and resistance pattern in bovine mastitis. Intas Polivet. 2015; 16(2):249-51.
188. Sargeant JM, Scott HM, Leslie KE, Ireland MJ, Bashiri A. Clinical mastitis in dairy cattle in Ontario: frequency of occurrence and bacteriological isolates. Can Vet J. 1998; 39(1):33-8.
189. Sayed HR, Salama SS, Soliman TR. Bacteriological evaluation of present situation of mastitis in dairy cows. Glob Vet. 2014; 13(5):690-5.
190. Schrick FN, Hockett ME, Saxton AM, Lewis MJ, Dowlen HH, Oliver SP. Influence of subclinical mastitis during early lactation on reproductive parameters. J Dairy Sci. 2001; 84(6):1407-12.
191. Seid U, Zenebe T, Almaw G, Edao A, Disassa H, Kabeta T, Gerbi F, Kebede G. Prevalence, risk factors and major bacterial causes of bovine mastitis in west Arsi zone of Oromia region, southern Ethiopia. Nat Sci. 2015; 13(8):19-27.
192. Sharma A, Chhabra R, Sindhu N. Prevalence of sub clinical mastitis in cows: Its etiology and antibiogram. Indian J Anim Res. 2012; 46(4):348-53.
193. Sharma A, Chhabra R, Singh M, Charaya G. Prevalence, etiology and antibiogram of bacterial isolates recovered from mastitis of buffaloes. Buffalo Bull. 2018; 37(3):313-20.
194. Sharma A, Dhingra P, Pander BL, Kumar R. Bovine sub clinical mastitis: prevalence and treatment with homeopathic medicine. Int J Cow Sci. 2006; 2(1):40-4.
195. Sharma A, Sindhu N. Occurrence of clinical and subclinical mastitis in buffaloes in the state of Haryana (India). Ital J Anim Sci. 2007; 6(2):965-7.
196. Sharma DK, Jallewar PK, Sharma KK. Antibiogram of bacteria isolated from bovine subclinical mastitis. Indian Vet J. 2010; 87(4):407.
197. Sharma I, Brinty A. Isolation and identification of staphylococcus aureus from bovine mastitis milk and their drug resistance patterns in Silchar town dairy farms, NE India. Int Interdiscip Res J. 2014; 4:256-9.
198. Sharma L, Verma AK, Kumar A, Rahat A, Neha, Nigam R. Incidence and pattern of antibiotic resistance of *Staphylococcus aureus* isolated from clinical and subclinical mastitis in cattle and buffaloes. Asian J Anim Sci. 2015; 9(3):100-9.

199. Sharma N, Maiti SK, Sharma KK. Prevalence, etiology and antibiogram of microorganisms associated with sub-clinical mastitis in buffaloes in Durg, Chhattisgarh State (India). Int J Dairy Sci. 2007; 2(2):145-51.
200. Shiferaw J, Telila I. Prevalence of bovine mastitis and assessment of risk factors in and around Wolayta Sodo, Ethiopia. Int J Hom Nat Med. 2016; 2(1):1-7.
201. Shitandi A, Kihumbu G. Assessment of the California mastitis test usage in smallholder dairy herds and risk of violative antimicrobial residues. J Vet Sci. 2004; 5(1):5-10.
202. Shome BR, Das MS, Bhuvana M, Krithiga N, Velu D, Shome R, Isloor S, Barbuddhe SB, Rahman H. Multiplex PCR assay for species identification of bovine mastitis pathogens. J Appl Microbiol. 2011; 111(6):1349-56.
203. Shrestha S, Bindari YR. Prevalence of sub-clinical mastitis among dairy cattle in Bhaktapur District, Nepal. Int J Agri Biosci. 2012; 1(1):16-9.
204. Silva NCC, Guimarães FF, Manzi MP, Budri PE, Gómez-Sanz E, Benito D, Langoni H, Rall VLM, Torres C. Molecular characterization and clonal diversity of methicillin-susceptible *Staphylococcus aureus* in milk of cows with mastitis in Brazil. J Dairy Sci. 2013; 96(11):6856-62.
205. Sindhu N, Sharma A, Chhabra R, Pankaj R. Prevalence of sub clinical mastitis in cows: Its etiology and antibiogram. Indian J Anim Res. 2012; 46(4):348-53.
206. Sindhu N, Sharma A, Nehra V, Jain VK. Occurrence of subclinical mastitis in cows and buffaloes at an organized farm. Haryana Vet. 2009; 48:85-7.
207. Singh M, Garg A. Incidence of different pathogens and milk compositional changes in sub-clinical and clinical milk samples of cows. Indian J Anim Res. 2011; 45(1):51-5.
208. Singh N, Singh P, Patel RK. Isolation and identification of bacterial organisms from mastitic milk. J Livest Sci. 2016; 7:46-8.
209. Singh PK, Diwakar RP, Johri A. Study the prevalence of *S. aureus* in subclinical mastitis and their antibiogram in buffaloes. J Pharmacogn Phytochem. 2018; 4:440-2.
210. Soltau JB, Einax E, Klengel K, Katholm J, Failing K, Wehrend A, Donat K. Within-herd prevalence thresholds for herd-level detection of mastitis pathogens using multiplex real-time PCR in bulk tank milk samples. J Dairy Sci. 2017; 100(10):8287-95.
211. Sori H, Zerihun A, Abdicho S. Dairy cattle mastitis in and around Sebeta, Ethiopia. Int J Appl Res Vet Med. 2005; 3(4):332.
212. Srinivasan P, Jagadeswaran D, Manoharan R, Giri T, Balasubramaniam GA, Balachandran P. Prevalence and etiology of subclinical mastitis among buffaloes (*Bubalus bubalus*) in Namakkal, India. Pak J Biol Sci. 2013; 16(23):1776-80.
213. Sudhakar PA, Khode NV, Sardar VM, Mendhe MS. Prevalence and current antibiogram trend of mastitic agents in Udgir and its vicinity, Maharashtra State, India. Int J Dairy Sci. 2009; 4(3):117-22.
214. Sudhan NA, Singh R, Singh M, Soodan JS. Studies on prevalence, etiology and diagnosis of subclinical mastitis among crossbred cows. Indian J Anim Res. 2005; 39(2):127-30.
215. Sudhanthiramani S, Swetha CS, Bharathy S. Prevalence of antibiotic resistant *Staphylococcus aureus* from raw milk samples collected from the local vendors in the region of Tirupathi, India. Vet World. 2015; 8(4):478.
216. Suleiman TS, Karimuribo ED, Mdegela RH. Prevalence of mastitis in smallholder dairy cattle in Pemba Island, Tanzania. Tanzan Vet J. 2013; 28:70-81.
217. Suleiman TS, Karimuribo ED, Mdegela RH. Prevalence of bovine subclinical mastitis and antibiotic susceptibility patterns of major mastitis pathogens isolated in Unguja island of Zanzibar, Tanzania. Trop Anim Health Pro. 2018; 50(2):259-66.
218. Suriyasathaporn W. Epidemiology of subclinical mastitis and their antibacterial susceptibility in smallholder dairy farms, Chiang Mai province, Thailand. J Anim Vet Adv. 2011; 10(3):316-21.

219. Swamy MCM, Krishnamurthy GV. Prevalence of Staphylococcus species in California mastitis test positive cows. Indian Vet J. 1998; 75(2):101-3.
220. Swarnakar G, Deepak S, Hardik G. Biofilm formation, hemolysin production and antimicrobial susceptibilities of *Staphylococcus aureus* isolated from the mastitis milk of buffaloes in Udaipur, India. Int J Vet Sci. 2017; 6(1):1-6.
221. Swinkels JM, Cox P, Schukken YH, Lam TJGM. Efficacy of extended cefquinome treatment of clinical *Staphylococcus aureus* mastitis. J Dairy Sci. 2013; 96(8):4983-92.
222. Sylejmani D, Ramadani N, Robaj A, Hamidi A. Prevalence and antimicrobial susceptibility of bacterial isolates from subclinical mastitis in dairy farms in Kosovo. Bulg J Vet Med. 2016; 19(4):299-307.
223. Szczubial M, Dabrowski R, Kankofer M, Bochniarz M, Komar M. Concentration of serum amyloid A and ceruloplasmin activity in milk from cows with subclinical mastitis caused by different pathogens. Pol J Vet Sci. 2012; 15(2):291-6.
224. Tadesse A, Chanie M. Study on the occurrence of bovine mastitis in Addis Ababa dairy farms and associated risk factors. Adv Biol Res. 2012; 6(4):151-8.
225. Tekle Y, Berihe T. Bovine mastitis: prevalence, risk factors and major pathogens in Sidama zone SNNPRS, Ethiopia. Int J Innov Sci Math. 2015; 3(5):230-8.
226. Tesfaye B. Bovine mastitis: Prevalence, risk factors, major pathogens and antimicrobial susceptibility test on the isolates around addis Ababa, Central Ethiopia. Int J Vet Sci Res. 2017; 2(5):1-6.
227. Tesfaye B, Abera A. Prevalence of mastitis and associated risk factors in Jimma Town Dairy Farms, Western Ethiopia. J Vet Sci Anim Hus. 2018; 6(3):1-8.
228. Thakor D, Patel D. Prevalence and antibiogram pattern of subclinical mastitis in crossbred cows, Natural Remedies Private Limited. https://en.engormix.com/dairy-cattle/articles/prevalence-antibiogram-pattern-sub-t35804.htm. 2013.
229. Thakur S, Singh M, Aseri GK, Verma A, Khan SS. Isolation and characterization of mastitis pathogens and milk composition changes in Murrah buffaloes (*Bubalus bubalis*) during winter season. Indian J Anim Res. 2018; 52(2):276-80.
230. Tripathi S, Arora N, Shekhar S, Rajora VS. Etio-prevalence of sub clinical mastitis in crossbred cattle. J Entomol Zool. 2018; 6(1):778-80.
231. Tufani NA, Makhdoomi DM, Hafiz A. Epidemiology and therapeutic management of bovine mastitis. Indian J Anim Res. 2012; 46(2):148-151.
232. Turutoglu H, Mudul S, Pehlivanoglu F. Antibiotic susceptibility and (3-lactamase prevalence for Staphylococci isolated from bovine mastitic milk samples. Acta Vet. 2002; 52(5-6):337-44.
233. Umar S, Sarwar F, Usman M, Shah MAA, Ghafar A, Ali A, Asif S. In vitro antimicrobial sensitivity pattern of mastitis causing bacterial pathogens isolated from cattle in arid zones of Punjab. Pakistan. Sci Lett. 2013; 1(1):17-20.
234. Umaru GA, Kwaga JKP, Bello M, Raji MA, Maitala YS. Occurrence of bovine mastitis and isolation of Staphyloccocus species from fresh cow milk in settled Fulani herds in Kaduna State. Nigeria. Bayero J Pure Appl Sci. 2017; 10(1):259-63.
235. Vakkamäki J, Taponen S, Heikkilä AM, Pyörälä S. Bacteriological etiology and treatment of mastitis in finnish dairy herds. Acta Vet Scand. 2017; 59(1):1-9.
236. Verma H, Rawat S, Sharma N, Jaiswal V, Singh R. Prevalence, bacterial etiology and antibiotic susceptibility pattern of bovine mastitis in Meerut. J Entomol Zool. 2018; 6:706-9.
237. Vikova H, Babak V, Vrtkova I, Cervinkova D, Marosevic D, Moravkova M, Jaglic Z. Epidemiology of intramammary infections with *Staphylococcus aureus* and mastitis Streptococci in a dairy cattle herd with a history of recurrent clinical mastitis. Pol J Vet Sci. 2017; 20(1):133-9.

238. Wanasinghe DD, Frost AJ. The prevalence of udder infection and mastitis in herds producing bulk milk with either consistently high or low cell count. Aust Vet J. 1979; 55(8):374-80.
239. Watson DL, McColl ML, Davies HI. Field trial of a staphylococcal mastitis vaccine in dairy herds: clinical, subclinical and microbiological assessments. Aust Vet J. 1996; 74(6):447-50.
240. Wilson DJ, Gonzalez RN, Das HH. Bovine mastitis pathogens in New York and Pennsylvania: prevalence and effects on somatic cell count and milk production. J Dairy Sci. 1997; 80(10):2592-8.
241. Wubishet Z, Ararsa D, Alemayehu L. Bovine mastitis in selected districts of Borena zone, southern Ethiopia. Bull Anim Health Prod Afr. 2013; 61(2):173-9.
242. Yohannes K, Alemu B. Prevalence of bovine mastitis in lactating cows and associated risk factors in and around Wolayta Soddo, southern Ethiopia. Int J Adv Res Biol Sci. 2018; 5(12):60-9.
243. Yohannis M, Molla W. Prevalence, risk factors and major bacterial causes of bovine mastitis in and around Wolaita Sodo, southern Ethiopia. Afr J Microbiol Res. 2013; 7(48):5400-5.
244. Zaki MS, El-Battrawy N, Mostafa SO. Some biochemical studies on friesian suffering from subclinical mastitis. Nat Sci. 2010; 8(4):143-6.
245. Zenebe N, Habtamu T, Endale B. Study on bovine mastitis and associated risk factors in Adigrat, northern Ethiopia. Afr J Microbiol Res. 2014; 8(4):327-31.
246. Zeryehun T, Abera G. Prevalence and bacterial isolates of mastitis in dairy farms in selected districts of eastern Harrarghe zone, eastern Ethiopia. J Vet Med. 2017; 2017:1-7.
247. Zeryehun T, Aya T, Bayecha R. Study on prevalence, bacterial pathogens and associated risk factors of bovine mastitis in small holder dairy farms in and around Addis Ababa, Ethiopia. J Anim Plant Sci. 2013; 23(1):50-5.
248. Zhang S, Piepers S, Shan R, Cai L, Mao S, Zou J, Ali T, De Vliegher S, Han B. Phenotypic and genotypic characterization of antimicrobial resistance profiles in *Streptococcus dysgalactiae* isolated from bovine clinical mastitis in 5 provinces of China. J Dairy Sci. 2018; 101(4):3344-55.
249. Zutic M, Cirkovic I, Pavlovic L, Zutic J, Asanin J, Radanovic O, Pavlovic N. Occurrence of methicillin-resistant *Staphylococcus aureus* in milk samples from Serbian cows with subclinical mastitis. Afr J Microbiol Res. 2012; 6(29):5887-9.
